# Integrative taxonomy of *Metrichia* Ross (Trichoptera: Hydroptilidae: Ochrotrichiinae) microcaddisflies from Brazil: descriptions of twenty new species

**DOI:** 10.7717/peerj.2009

**Published:** 2016-05-05

**Authors:** Allan P.M. Santos, Daniela M. Takiya, Jorge L. Nessimian

**Affiliations:** 1 Departamento de Zoologia, Universidade Federal do Estado do Rio de Janeiro, Rio de Janeiro, Brazil; 2 Departamento de Zoologia, Universidade Federal do Rio de Janeiro, Rio de Janeiro, Brazil

**Keywords:** Caddisfly, Integrative taxonomy, DNA barcoding, Aquatic insects, Neotropics

## Abstract

*Metrichia* is assigned to the Ochrotrichiinae, a group of almost exclusively Neotropical microcaddisflies. *Metrichia* comprises over 100 described species and, despite its diversity, only one species has been described from Brazil so far. In this paper, we provide descriptions for 20 new species from 8 Brazilian states: *M. acuminata* **sp. nov.**, *M. azul* **sp. nov.**, *M. bonita* **sp. nov.**, *M. bracui* **sp. nov.**, *M. caraca* **sp. nov.**, *M. circuliforme* **sp. nov.**, *M. curta* **sp. nov.**, *M. farofa* **sp. nov.**, *M. forceps* **sp. nov.**, *M. formosinha* **sp. nov.**, *M. goiana* **sp. nov.**, *M. itabaiana* **sp. nov.**, *M. longissima* **sp. nov.**, *M. peluda* **sp. nov.**, *M. rafaeli* **sp. nov.**, *M. simples* **sp. nov.**, *M. talhada* **sp. nov.**, *M. tere* **sp. nov.**, *M. ubajara* **sp. nov.**, and *M. vulgaris* **sp. nov.** DNA barcode sequences (577 bp of the mitochondrial gene COI) were generated for 13 of the new species and two previously known species of *Metrichia* resulting in 64 sequences. In addition, COI sequences were obtained for other genera of Ochrotrichiinae (*Angrisanoia*, *Nothotrichia*, *Ochrotrichia*, *Ragatrichia*, and *Rhyacopsyche*). DNA sequences and morphological data were integrated to evaluate species delimitations. K2P pairwise distances were calculated to generate a neighbor-joining tree. COI sequences also were submitted to ABGD and GMYC methods to assess ‘potential species’ delimitation. Analyses showed a conspicuous barcoding gap among *Metrichia* sequences (highest intraspecific divergence: 4.8%; lowest interspecific divergence: 12.6%). Molecular analyses also allowed the association of larvae and adults of *Metrichia bonita* **sp. nov.** from Mato Grosso do Sul, representing the first record of microcaddisfly larvae occurring in calcareous tufa (or travertine). ABGD results agreed with the morphological delimitation of *Metrichia* species, while GMYC estimated a slightly higher number of species, suggesting the division of two morphological species, each one into two potential species. Because this could be due to unbalanced sampling and the lack of morphological diagnostic characters, we have maintained these two species as undivided.

## Introduction

The microcaddisfly genus *Metrichia* Ross, 1938 is included in the subfamily Ochrotrichiinae, which also includes *Ochrotrichia* Mosely, 1934, *Angrisanoia* Özdikmen, 2008, *Nothotrichia* Flint, 1967, *Rhyacopsyche* Müller, 1879, and the recently erected *Ragatrichia* Oláh & Johanson, 2011, all of them exclusively from New World. Based on adult morphology, Harris & Armitage (1997) and Oláh & Johanson (2011) also suggested three other genera to be included in this subfamily: *Dibusa* Ross, 1939, *Caledonotrichia* Sykora, 1967, and *Maydenoptila* Neboiss, 1977, from the USA, New Caledonia, and Australia, respectively. However, because diversification of main lineages of hydroptilids has not been deeply studied, the placement of these genera remains dubious. As noted by Wells, Johanson & Mary-Sasal (2013), relationships of these microcaddisflies need to be studied based on rigorous analyses including molecular data.

Currently, *Metrichia* includes 107 species, found from the USA to South America, with highest known diversity in Central America (Flint Jr, 1972; Marshall, 1979). *Metrichia* was considered as a subgenus of *Ochrotrichia* due to similarities of adult morphology and almost indistinguishable larvae (Flint Jr, 1968). This subgeneric status was followed by Marshall (1979), who also established the New World tribe that is now recognized as subfamily Ochrotrichiinae. Wiggins (1996) provided additional information on larval morphology of *Metrichia* and *Ochrotrichia*, reestablishing both as independent genera.

**Figure 1:**
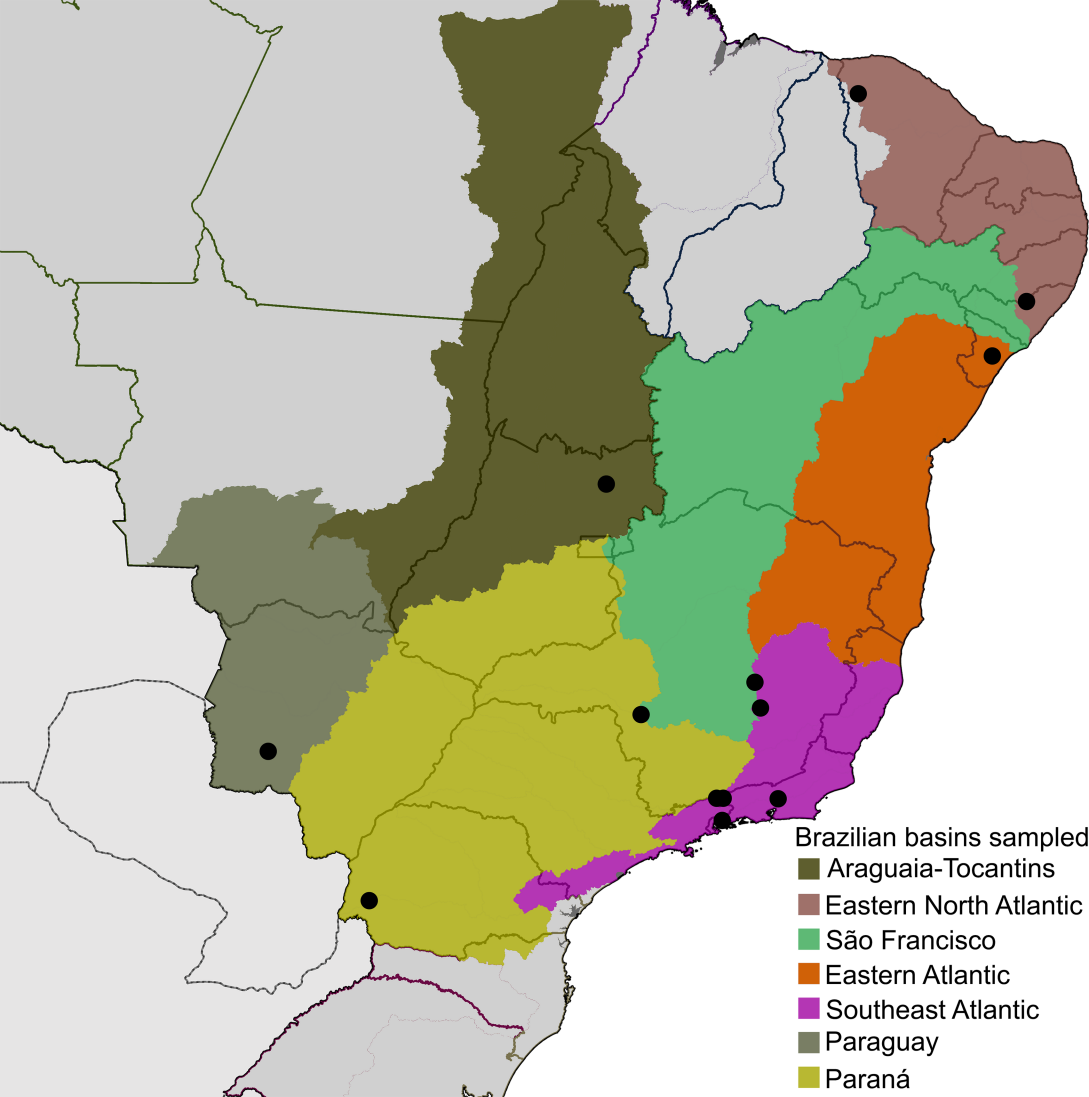
Localities (●), distributed on seven of the large river basins of Brazil, where *Metrichia* specimens studied herein were collected.

Diversity of Neotropical microcaddisflies is poorly known and usually several undescribed species are found in collections or amongst recently collected material when examined by experts, even in localities previously studied by trichopterologists. This likely occurs because Hydroptilidae are very small and have complex male genitalia, making them difficult to observe by lower magnification microscopes and to understand homologies among some structures. Only one species of *Metrichia* has been described from Brazil so far, *M*. *pernambucana* Souza & Santos, 2013, but larvae have been commonly identified from several localities (e.g., Pes, Hamada & Nessimian, 2005; Spies & Froehlich, 2009; Spies, Froehlich & Kotzian, 2006). It is not surprising that material studied herein recently collected from different river basins in Brazil (Fig. 1, Supplemental Information 1 and 2) revealed so many undescribed species.

Most *Metrichia* species exhibit a more restricted distribution, in other words, each species has been recorded only from type-localities or relatively few close localities. Herein we describe one species with a wide distribution in Brazil, occurring in three very distinctive biomes: Atlantic Forest (Southeastern Brazil), Caatinga (Northeastern Brazil), and Cerrado (Centralwestern Brazil). Although such wide distributions are not common in *Metrichia*, similar examples are known for other Neotropical microcaddisflies, as *Oxyethira tica* Holzenthal & Harris, 1992, recorded from Mexico, Central, and South America (Flint Jr, Holzenthal & Harris, 1999).

Although molecular tools have become common in taxonomic studies to help in species delimitation, their use is still rare with Neotropical caddisflies. Using sequences of the mitochondrial cytochrome oxidase I gene (COI), the standard DNA barcode region for animals (Hebert et al., 2003), Pauls et al. (2010) were able to corroborate two new species of *Smicridea* (*Smicridea*) McLachlan, 1871 from Chile, defined with morphological characters. In most studies with caddisflies, divergence in COI sequences has shown clear differences between intraspecific and interspecific variation, the so-called ‘barcoding gap’ (Zhou, Kjer & Morse, 2007; Pauls et al., 2010; Ruiter, Boyle & Zhou, 2013). Some species delimitation approaches rely solely in distinguishing intra- and interspecific divergence, such as the Automatic Barcoding Gap Discovery (ABGD) (Puillandre et al., 2012). More sophisticated methods invoke coalescence and speciation models, such as the General Mixed Yule Coalescent (GMYC), and are considered more robust for identifying lineages when intra- and interspecific divergences overlap (Pons et al., 2006). Integrating independent data (e.g., morphology and DNA sequences) and using different approaches are particularly interesting for the taxonomy of diverse and complex groups, such as microcaddisflies. In this work, we applied both ABGD and GMYC methodologies to evaluate our initial morphological identification.

Besides its use for species delimitation, DNA taxonomy has a valuable role in making associations between immature and adult stages of caddisflies (Graf, Lubini & Pauls, 2005; Waringer et al., 2007; Zhou, Kjer & Morse, 2007; Ruiter, Boyle & Zhou, 2013). Traditional techniques to associate larvae and adults are more difficult because they involve rearing larvae in the laboratory (not an easy task for many caddisfly groups) or luck in finding pharate adults in field. Indirect association of stages, for example, by collecting adults and larvae at the same locality, can result in misidentification, because different species of the same genus frequently co-occur.

Larvae of *Metrichia* have been associated for only two species: *M*. *nigritta* (Banks, 1907) described by Edwards & Arnold (1961) and illustrated by Wiggins (1996), and *M*. *juana* (Flint Jr, 1964) by Flint Jr (1964) in the original description. In addition, Botosaneanu & Flint Jr (1982) described a larva of *Metrichia* and its case from Venezuela and a pupal case from Ecuador; and Pes, Hamada & Nessimian (2005) illustrated larvae from Brazil and three different types of cases. In both works, specific names were not provided as authors did not have respective adults. *Metrichia* larvae build a typical oval purse-like case, made of silk, usually covered with algae filaments (Wiggins, 1996; Pes, Hamada & Nessimian, 2005), and sometimes also having sand grains (Botosaneanu & Flint Jr, 1982). Cases of some larvae that have not yet been associated with adults show a pair of dorsal “chimneys,” an uncommon feature also described and illustrated by Müller (1879) and Müller (1880) for *Dicaminus ladislavii* Müller, 1879, from Santa Catarina, Brazil. Based on larval cases from Central and South America, Botosaneanu & Flint Jr (1982) pointed that *Dicaminus* is possibly synonymous with *Metrichia*, but this question remains open, since there are no male specimens from Müller’s work.

Almost nothing is known about the biology of *Metrichia* larvae. According to Wiggins (1996), larvae of *M*. *nigritta* were collected in association with filamentous algae on rock surfaces. In Brazil, *Metrichia* occurs in fast flowing streams, usually with associated algae. Herein, we report for the first time the occurrence of *Metrichia* in calcareous tufa. Calcareous tufa or travertine is a terrestrial sedimentary rock, predominantly composed of carbonate minerals, calcite, and aragonite (Drysdale, 1998). Travertines are formed by rapid precipitation of these minerals, producing large alterations on river morphology (Drysdale & Gale, 1997). Although the importance of microbes on travertine formation is recognized by creating surfaces for crystal nucleation, our knowledge of the importance of macroinvertebrates in this process is still poor (Drysdale, 1998; Drysdale, 1999; Paprocki, Holzenthal & Cressa, 2003). Studies by Drysdale (1998) and Drysdale (1999) pointed out that aquatic insects play an important role in travertine biogenesis in Australian springs, especially *Cheumatopsyche* Wallengreen, 1891 (Hydropsychidae) larvae. Paprocki, Holzenthal & Cressa (2003) also found another Hydropsychidae (*Smicridea*) to be an important organism in modifying travertine morphology in Venezuela.

## Material and Methods

### Morphological study

Specimens were collected manually (larvae or diurnal active adults) or using Malaise or light traps, and then fixed in 96% ethanol. Collecting permits in Brazil were issued by Instituto Chico Mendes de Conservação da Biodiversidade (ICMBio) (SISBIO 43047 and 14591). To observe genital structures, abdomen of males were removed and cleared in a heated solution of 10% KOH for 20 min. Then, abdomens were mounted in temporary slides, which were used to draw pencil sketches with compound microscope equipped with camera lucida. Vector graphics were traced in Adobe Illustrator CS6 (Adobe Systems Inc.) using pencil sketches as templates. Descriptions provided here were made with DELTA software (Description Language for Taxonomy) (Dallwitz, Paine & Zurcher, 1999). Terminology used throughout this paper follows that provided by Marshall (1979) and Bueno-Soria & Holzenthal (2003). Types for newly described species are deposited at Coleção Entomológica Prof. José Alfredo Pinheiro Dutra, Departamento de Zoologia, Universidade Federal do Rio de Janeiro, Rio de Janeiro (DZRJ); Museu Nacional, Universidade Federal do Rio de Janeiro, Rio de Janeiro (MNRJ); Instituto Nacional de Pesquisas da Amazônia, Manaus (INPA); Coleção Zoológica do Maranhão (CZMA), Caxias; and Museu de Zoologia da Universidade Federal da Bahia, Salvador (MZUFBA).

The electronic version of this article in Portable Document Format (PDF) will represent a published work according to the International Commission on Zoological Nomenclature (ICZN), and hence the new names contained in the electronic version are effectively published under that Code from the electronic edition alone. This published work and the nomenclatural acts it contains have been registered in ZooBank, the online registration system for the ICZN. The ZooBank LSIDs (Life Science Identifiers) can be resolved and the associated information viewed through any standard web browser by appending the LSID to the prefix http://zoobank.org/. The LSID for this publication is: urn:lsid:zoobank.org:pub:D8D4049E-494B-4A30-92AC-F8F42D2B54B9. The online version of this work is archived and available from the following digital repositories: PeerJ, PubMed Central and CLOCKSS.

### DNA extraction, PCR, and sequencing

Genomic DNA was extracted from head and thorax (or from the entire body) of fresh material using the DNeasy Blood and Tissue Kit (QIAGEN, Hilden, Germany), without tissue maceration. After extraction, specimens were returned to ethanol and deposited in DZRJ collection as a DNA voucher. COI fragments were amplified using pair of primers: HCO-2198 (5′-TAAACTTCAGGGTGACCAAAAAATCA-3′) in combination with LCO-1490 (5′-GGTCAACAAATCATAAAGATATTGG-3′) (Folmer et al., 1994) or C1-J-1718 (5′-GGAGGATTTGGAAATTGATTAGTTCC-3′) (Simon et al., 1994). Polymerase chain reaction (PCR) conditions were as follows: initial denaturation at 94 °C for 3 min; 35 cycles of denaturation at 94 °C for 1 min, annealing at 50 °C for 1 min, and extension at 72 °C for 2 min; and final extension at 72 °C for 7 min. PCR products were sent to Macrogen Inc., Seoul, for purification and sequencing reactions.

COI sequences of 64 specimens of 15 species of *Metrichia* were obtained. Additional sequences were obtained for specimens of *Angrisanoia*, *Nothotrichia*, *Ochrotrichia*, and *Rhyacopsyche* (Table 1), included as outgroup in different analyses, as described below.

**Table 1:** Species of *Metrichia* and other hydroptilids with DNA barcodes sequenced and used in this study, with respective information of specimen voucher and GenBank Accession Numbers.

Species	Voucher code and life stage	Collection site	GenBank accession number
*Angrisanoia cebollati* (Angrisano, 1995)	ENT 2199 ♂	Brazil: Goiás: Alto Paraíso de Goiás	–
*Betrichia bispinosa* Flint, 1974	ENT 2337 ♂	Brazil: Amapá	KU094961^b^
*Nothotrichia cautinensis* Flint, 1983	–	–	KC559534^a^
*Nothotrichia tupi* Holzenthal & Harris, 1992	ENT 2460 ♂	Brazil: Minas Gerais: Catas Altas	KU743400
*Ochrotrichia caatinga* Souza, Santos & Takiya, 2014	ENT 2472 ♂	Brazil: Ceará: Ubajara	KU743401
*Ochrotrichia patulosa* (Wasmund & Holzenthal, 2007)	ENT 2473 ♂	Brazil: Ceará: Ubajara	KU743402
*Ochrotrichia* sp. CR1	ENT 2279 ♂	Costa Rica: Puntarenas	KU094950^b^
*Oxyethira tica* Holzenthal & Harris, 1992	ENT 0057 ♂	Brazil: Pará: Carajás	KU094940^b^
*Ragatrichia* sp. BR1	ENT 2338 ♂	Brazil: Amapá	KU743403
*Rhyacopsyche dikrosa* Wasmund & Holzenthal, 2007	ENT 0122 ♂	Brazil: Rio de Janeiro: Teresópolis	KU094952^b^
*Rhyacopsyche torulosa* Flint, 1971	ENT 2277 ♂	Costa Rica: Puntarenas	KU743404
*Metrichia acuminata* **sp. nov.**	ENT 2192 ♂	Brazil: Alagoas: Quebrangulo	KU743406
	ENT 2282 ♂		KU743427
	ENT 2284-5 ♂		KU743428– KU743429
	ENT 2779 ♂		KU743452
*Metrichia amplitudinis* Bueno-Soria & Holzenthal, 2003	ENT 2278 ♂	Costa Rica: Puntarenas	KU743425
*Metrichia bonita* **sp. nov.**	ENT 2200-4 larvae	Brazil: Mato Grosso do Sul: Bonito	KU743409, KU743410, KU743411, KU743412, KU743413
*Metrichia bonita* **sp. nov.**	ENT 2208-10 ♂	Brazil: Mato Grosso do Sul: Bonito	KU743417, KU743418, KU743419
*Metrichia bracui* **sp. nov.**	ENT 2508-11 ♂	Brazil: Rio de Janeiro: Itatiaia	KU743444, KU743445, KU743446, KU743447
*Metrichia caraca* **sp. nov.**	ENT 2195 ♂	Brazil: Minas Gerais: Catas Altas	KU743408
	ENT 2280 ♂		KU743426
	ENT 2461-5 ♂		KU743434, KU743435, KU743436, KU743437, KU743438
*Metrichia caraca* **sp. nov.**	ENT 2292 ♂	Brazil: Minas Gerais: São Roque de Minas	KU743432
*Metrichia circuliforme* **sp. nov.**	ENT 2835-7 ♂	Brazil: Rio de Janeiro: Itatiaia	KU743455, KU743456, KU743457
	ENT 2839-40 ♂		KU743459– KU743460
	ENT 2843-4 ♂		KU743462– KU743463
*Metrichia curta* **sp. nov.**	ENT 2838 ♂	Brazil: Rio de Janeiro: Itatiaia	KU743458
	ENT 2846-8 ♂		KU743464, KU743465, KU743466
*Metrichia formosinha* **sp. nov.**	ENT 2205-7 ♂	Brazil: Mato Grosso do Sul: Bonito	KU743414, KU743415, KU743416
*Metrichia itabaiana* **sp. nov.**	ENT 2190 ♂	Brazil: Sergipe: Areia Branca	KU743405
*Metrichia itabaiana* **sp. nov.**	ENT 2220-1 ♂	Brazil: Goiás: Alto Paraíso de Goiás	KU743424
*Metrichia juana* (Flint, 1964)	ENT 2850-1 ♂	Puerto Rico	KU743467– KU743468
*Metrichia longissima* **sp. nov.**	ENT 2330 ♂	Brazil: Rio de Janeiro: Teresópolis	KU743433
*Metrichia longissima* **sp. nov.**	ENT 2841 ♂	Brazil: Rio de Janeiro: Itatiaia	KU743461
*Metrichia rafaeli* **sp. nov.**	ENT 2288-9 ♂	Brazil: Ceará: Ubajara	KU743430– KU743431
*Metrichia talhada* **sp. nov.**	ENT 2193 ♂	Brazil: Alagoas: Quebrangulo	KU743407
	ENT 2214 ♂		KU743420, KU743421, KU743422
	ENT 2216-7 ♂		KU743451
	ENT 2776 ♂		
*Metrichia tere* **sp. nov.**	ENT 2773-5 ♂	Brazil: Rio de Janeiro: Teresópolis	KU743448, KU743449, KU743450
*Metrichia vulgaris* **sp. nov.**	ENT 2218 ♂	Brazil: Goiás: Alto Paraíso de Goiás	KU743423
*Metrichia vulgaris* **sp. nov.**	ENT 2466-70 ♂	Brazil: Minas Gerais: Catas Altas	KU743439, KU743440, KU743441, KU743442, KU743443
*Metrichia vulgaris* **sp. nov.**	ENT 2833-4 ♂	Brazil: Rio de Janeiro: Itatiaia	KU743453– KU743454

**Notes.**

Sequences obtained from GenBank.

a Malm, Johanson & Wahlberg (2013).

b Santos, Nessimian & Takiya (2016).

### Sequence editing, alignment, and analyses

Forward and reverse sequences of each sample were assembled and manually edited in Sequencher 4.1 (Gene Codes, Ann Arbor, Michigan, USA). Sequences were verified with the Blast tool in GenBank to check for contamination. Subsequently, COI sequences were aligned with ClustalW implemented in MEGA 6 (Tamura et al., 2013) and translated into amino-acid sequences to check for stop codons. The final alignment resulted in a matrix with 577 bp (Supplemental Information 3).

COI sequences were used to explore putative species limits with four different methodologies: (1) lineages recovered in neighbor-joining tree; (2) lineages recovered with Bayesian Inference; (3) ABGD; and (4) GMYC. The neighbor-joining tree was calculated in MEGA 6 using Kimura 2-Parameter (K2P) distances (Kimura, 1980), with partial deletion of missing information. Although the use of K2P distances in DNA barcoding is debated (Srivathsan & Meier, 2012), to allow comparison with previous works we also used this evolutionary model because it is frequently used in studies of species delimitation based on COI sequences. Branch support of neighbor-joining tree was assessed with 1,000 pseudoreplicates of non-parametric bootstrap (Felsenstein, 1985).

BI analysis was conducted with MrBayes v. 3.2.2 (Ronquist et al., 2012) with four independent runs, each one with four MCMC chains running for 50,000,000 generations, with sample frequency of 5,000. Convergence of sampled parameters was checked in Tracer v. 1.5 (Rambaut & Drummond, 2007) and the first 10% of sampled trees and parameters discarded as burnin. GTR + I + G was the best fit model selected by Akaike Information Criterion (AIC) with jModeltest v. 0.1.1 (Posada, 2008) and it was applied in BI analysis in MrBayes. Branch support was assessed by posterior probability (PP), presented on a 50% majority consensus tree.

ABGD analysis was run using the on-line version available in http://wwwabi.snv.jussieu.fr/public/abgd/, where the COI alignment was uploaded. The analysis was conducted with the following settings: Pmin = 0.001; Pmax = 0.1; steps = 20; relative gap width = 1.0, also based on K2P model. This method statistically infers the DNA barcode gap in a single locus alignment, partitioning the data based on this gap in putative species (Puillandre et al., 2012).

The GMYC analysis (Pons et al., 2006; Fujisawa & Barraclough, 2013) was performed in R (R Development Core Team, 2010) using the SPLITS package (Ezard, Fujisawa & Barraclough, 2009) with single-threshold method. Basically, the method estimates branching patterns on an ultrametric tree, identifying the most likely transition point from coalescent to speciation branching. The ultrametric tree used here was obtained with BEAST v. 1.8 (Drummond et al., 2012) under a relaxed uncorrelated molecular clock (Drummond et al., 2006). The node including *Ochrotrichia* species was calibrated based on fossil evidence with a lognormal distribution offset at 20 mya and log(mean) = 2.8 to represent the possible range of 20–140 mya (Wells & Wichard, 1989); and the divergence of Ochrotrichiinae was calibrated based on Malm, Johanson & Wahlberg (2013) with a normal distribution with mean 82.17 ± 12 mya. The BEAST analysis ran for 200,000,000 generations, sampled every 10,000 generations. Convergence was verified with Tracer and a maximum credibility tree was written using TreeAnotator, discarding the first 10% as burnin.

## Results

**Figure 2:**
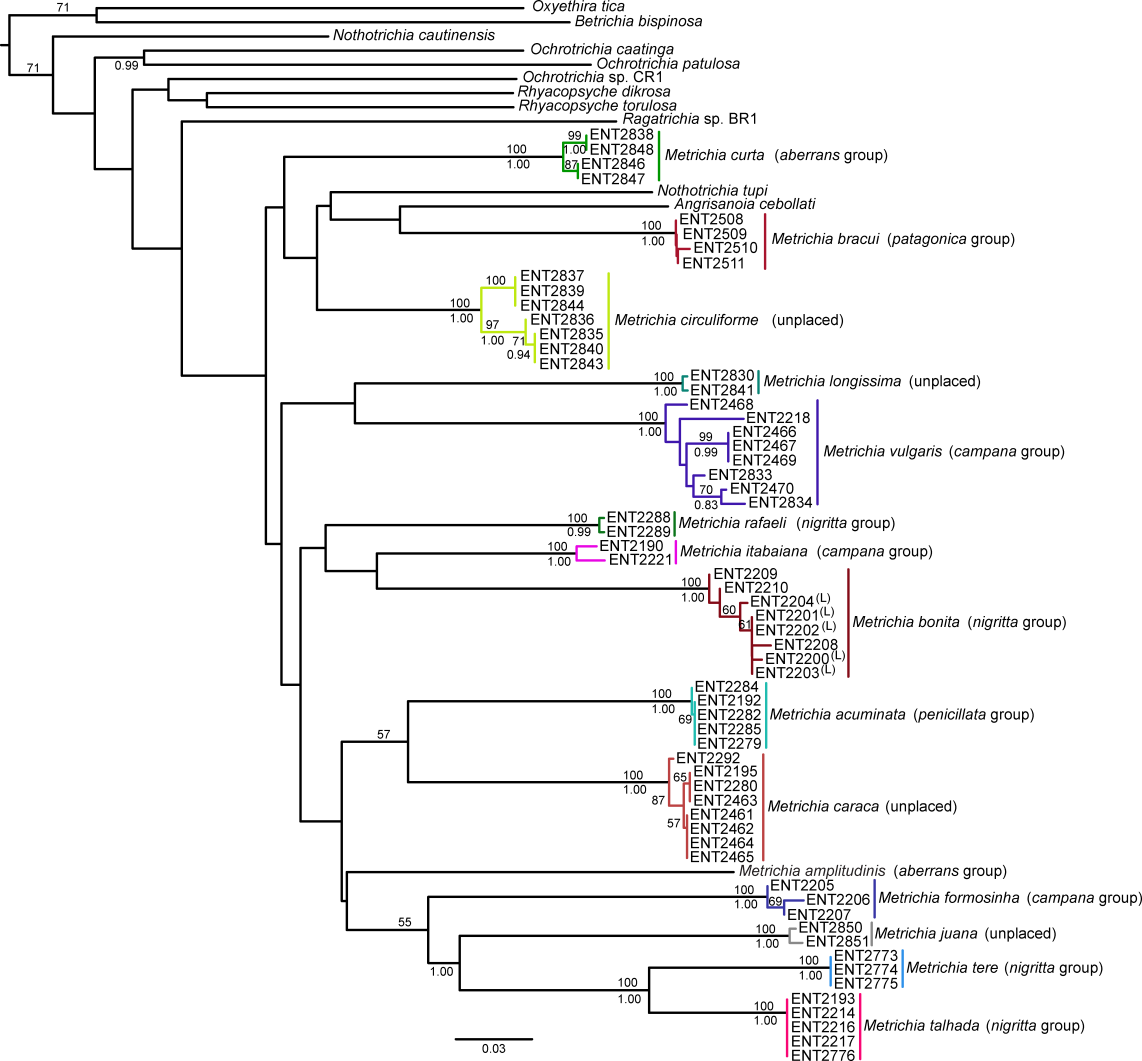
Neighbor-joining tree of COI sequences of *Metrichia* species based on K2P distances. Numbers above and below branches are, respectively, NJ bootstrap support and posterior probabilities from BI analysis. Details of specimens are in Tables 1 and 2; K2P distances matrix is in Supplemental Information 5.

**Figure 3:**
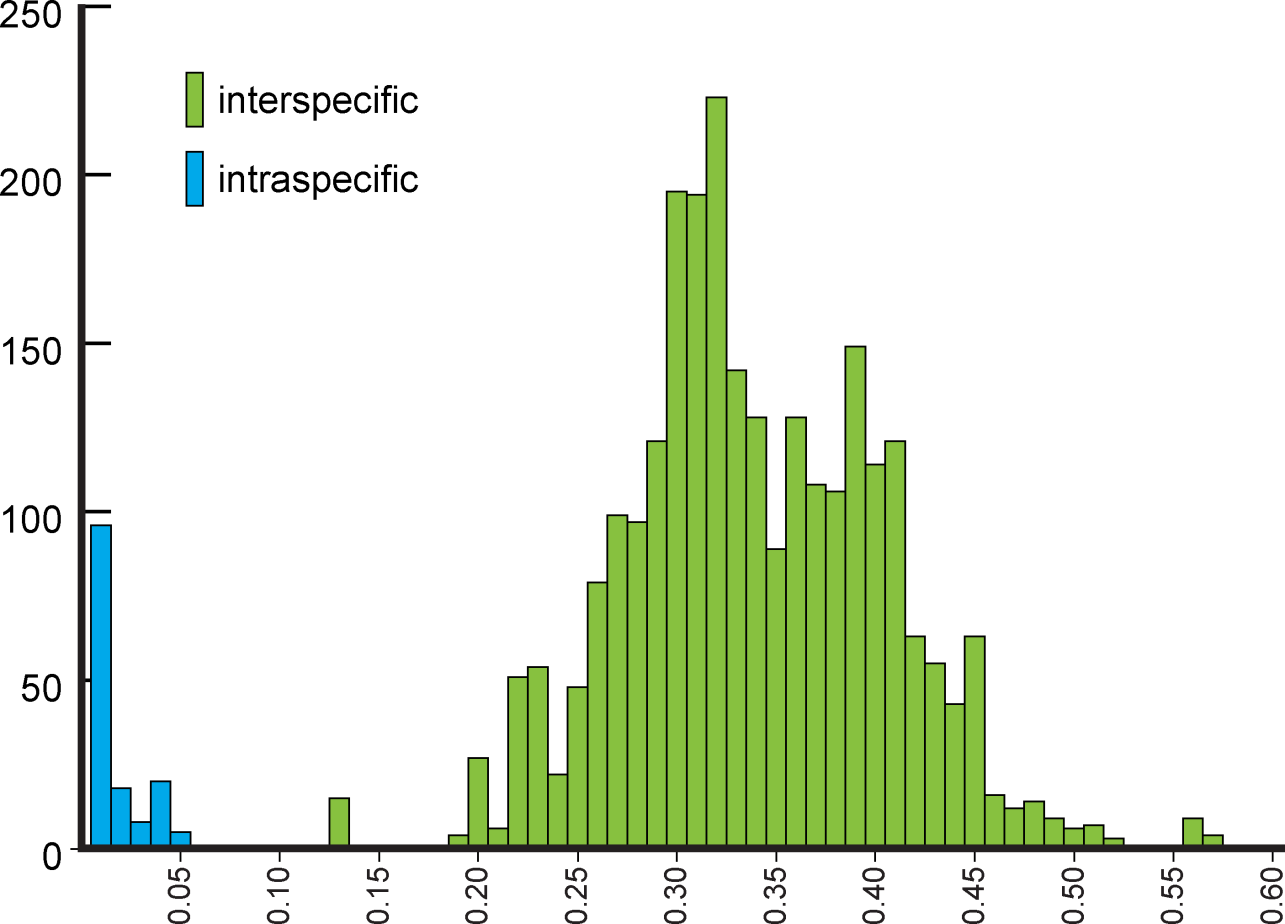
Histogram of the number of pairwise comparisons of intra- (blue) and interspecific (green) K2P divergences among 15 *Metrichia* species with COI sequences sampled.

NJ (Fig. 2) and BI (Supplemental Information 4) trees corroborated morphological identification, with all 14 species of *Metrichia* with more than a single specimen recovered as monophyletic lineages with 100% bootstrap support. ABGD also returned the same species as they were previously delimited based on morphological features. A robust ‘barcoding gap’ was found among *Metrichia* species (Fig. 3 and Table 2). The maximum intraspecific divergence was observed within *Metrichia vulgaris* **sp. nov.** (0.048). The minimum interspecific divergence was found between specimens of *Metrichia talhada* **sp. nov.** and *Metrichia tere* **sp. nov.** (0.126).

**Figure 4:**
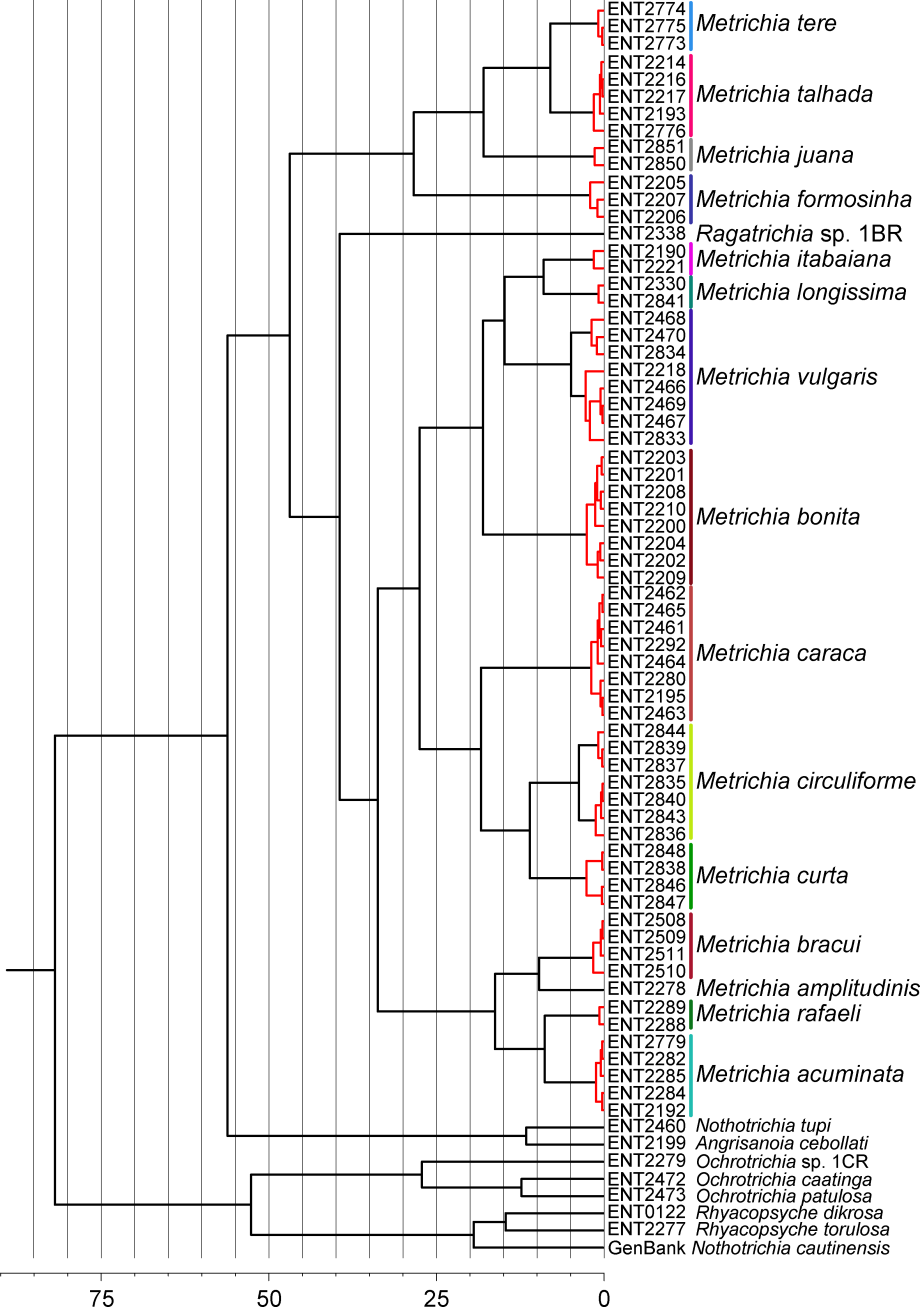
Maximum credibility time tree from the BEAST analysis based on COI sequences of *Metrichia* and other Ochrotrichiinae. Red branches represent species estimated by GMYC using single threshold in SPLITS. Timescale in millions of years.

GMYC analysis estimated a slightly higher number of putative species, with *Metrichia circuliforme* **sp. nov.** and *Metrichia vulgaris* **sp. nov.** being each further divided into two species (Fig. 4). Regarding all other species, GMYC results were congruent with other methods and with morphology.

**Table 2:** Maximum intra- and minimum interspecific K2P divergences of COI sequences among and within *Metrichia* species.

Species	Number of sequences	Max. intra. distance	Min. inter. distance
*M. acuminata* **sp. nov.**	5	0.000	0.217
*M*.*amplitudinis*Bueno-Soria & Holzenthal, 2003	1	–	0.220
*M*.*bonita* **sp. nov.**	8	0.015	0.210
*M*.*bracui* **sp. nov.**	4	0.004	0.214
*M. caraca* **sp. nov.**	8	0.011	0.217
*M*.*circuliforme* **sp. nov.**	7	0.035	0.184
*M*.*curta* **sp. nov.**	4	0.015	0.184
*M*.*formosinha* **sp. nov.**	3	0.008	0.249
*M*.*itabaiana* **sp. nov.**	2	0.019	0.194
*M*.*juana* (Flint, 1964)	2	0.007	0.243
*M*.*longissima* **sp. nov.**	2	0.004	0.215
*M*.*rafaeli* **sp. nov.**	2	0.004	0.194
*M*.*talhada* **sp. nov.**	5	0.000	0.126
*M*.*tere* **sp. nov.**	3	0.000	0.126
*M*.*vulgaris* **sp. nov.**	8	0.048	0.246

In all analyses performed using DNA barcode, *Metrichia* larvae collected in calcareous tufa were consistently associated with adult males of *Metrichia bonita* **sp. nov.** Therefore, in the following section, we describe these larvae within that species.

## Species Descriptions

### *Metrichia acuminata* sp. nov.

urn:lsid:zoobank.org:act:01493211-CD39-4995-B2D7-8F3B44A0970E

(Figs. 5 and 26A)

**Figure 5:**
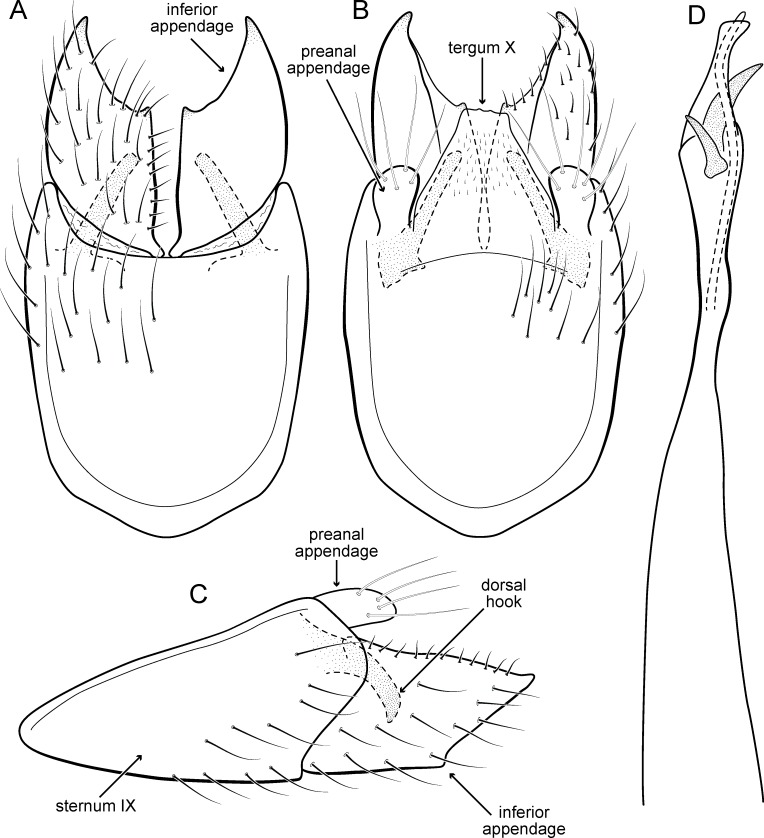
*Metrichia acuminata* **sp. nov.**, male genitalia. (A) ventral view; (B) dorsal view; (C) lateral view; (D) phallus, dorsal view.

**Adult male.** Length 2.1–2.5 mm (*n* = 5). General color, in alcohol, brown. Head with no modifications. Ocelli 3. Antenna simple, 18-articulated. Maxillary palpus 5-articulated; labial palpus 3-articulated. Mesoscutellum with transverse suture. Metascutellum subtriangular. Anterior femur without processes. Tibial spur formula 1-3-4. Wing venation reduced in both wings. Abdominal segment IV with pair of internal pouches in posterior area; segment V with pair of internal pouches; segment VI with tergum as a sclerotized rounded plate, surrounded by long setae (Fig. 26A); segment VII bearing a brush of very long setae dorsolateraly (Fig. 26A). Ventromesal process on segment VII present. Segment VIII shorter ventrally than dorsally and bearing a brush of long setae dorsally. **Male genitalia**. Segment IX reduced dorsally; sternum subrectangular, with anterior margin rounded (Fig. 5A); in lateral view narrower anteriorly than posteriorly (Fig. 5C). Inferior appendage covered by long setae; subtrapezoidal in ventral view (Fig. 5A); in lateral view, subtrapezoidal, apex with acute corners (Fig. 5C). Dorsal hook short, almost half length of inferior appendage; in lateral view, downturned (Fig. 5C). Preanal appendage rounded in lateral view and bearing very long setae (Fig. 5C). Subgenital plate apparently absent. Tergum X membranous and truncate (Fig. 5B). Phallus tubular, elongate and slender, slightly constricted mesally, with two curved subapical spines, one short and another long; apex truncate and sclerotized; ejaculatory duct sclerotized, sinuous, and protruding apically (Fig. 5D).

**Holotype.** **BRAZIL: Ceará:** Ubajara, Parque Nacional de Ubajara, Cachoeira do Gameleira, $03\textdegree 5{0}^{^{\prime}}2{1}^{^{\prime}^{\prime}}\mathrm{S}$ $40\textdegree 5{4}^{^{\prime}}2{3}^{^{\prime}^{\prime}}\mathrm{W}$, el. 880 m, 23.iv.2012, DM Takiya & JA Rafael cols., light trap, male (CZMA).

**Paratypes.** Same data as holotype, except, Rio das Minas, $03\textdegree 5{0}^{^{\prime}}0{3}^{^{\prime}^{\prime}}\mathrm{S}$ $40\textdegree 5{4}^{^{\prime}}1{8}^{^{\prime}^{\prime}}\mathrm{W}$, el. 524 m, 13–17.ix.2012, JA Rafael et al., Malaise trap, 2 males (INPA); same data, except 14–16.ii.2013, DM Takiya, JA Rafael, RR Cavichioli & APM Santos, Malaise trap, 2 males (DZRJ). **Alagoas:** Quebrangulo, Reserva Biológica de Pedra Talhada, Rio Caranguejo, $09\textdegree 1{5}^{^{\prime}}2{6}^{^{\prime}^{\prime}}\mathrm{S}$ $36\textdegree 2{5}^{^{\prime}}0{8}^{^{\prime}^{\prime}}\mathrm{W}$, el. 550 m, 19–28.vi.2014, APM Santos, DM Takiya, WRM Souza, Malaise trap, 2 males (MNRJ), 3 males (MZUFBA), 13 males (DZRJ).

**Etymology.** The species is named in allusion to the pointed apices of inferior appendages (from Latin, “acumin-” = “pointed”).

**Remarks**. This new species belongs to the *penicillata* group based on: (1) internal pouches between abdominal segments IV and V; (2) setal brushes on segments V, VI, and VII; and (3) phallus with two subapical spines. The male genitalia and complex abdominal modifications resemble *M*. *penicillata* (Flint Jr, 1972) and *M*. *trigonella* (Flint Jr, 1972). These three species have inferior appendages with acute apices in lateral view; phallus with two subapical spines; and abdominal terga with brushes of very long and stout setae. The new species can be distinguished by inferior appendages more trapezoidal in lateral view, with acute corners posteriorly and dorsal hook only slightly downturned in lateral view; and phallus with one larger and stouter subapical spine. Although the male genitalia of this new species superficially resemble that of *M*. *bonita* **sp. nov.**, *M*. *acuminata* **sp. nov.** is readily recognized by setose lobes on abdominal segments V and VI.

We were not able to obtain COI sequences for individuals from Ceará State, so the five sequences analyzed belong to specimens from Alagoas State, which shared the same haplotype. *Metrichia acuminata* **sp. nov.** was recovered as closely related to *M*. *caraca* **sp. nov.** (Fig. 2), but in both Bayesian approaches these two species were not recovered as sister taxa (Fig. 4, Supplemental Information 1).

### *Metrichia azul* sp. nov.

urn:lsid:zoobank.org:act:E38ACAE9-61DC-4B61-A20D-1879520E1DD3

(Fig. 6)

**Figure 6:**
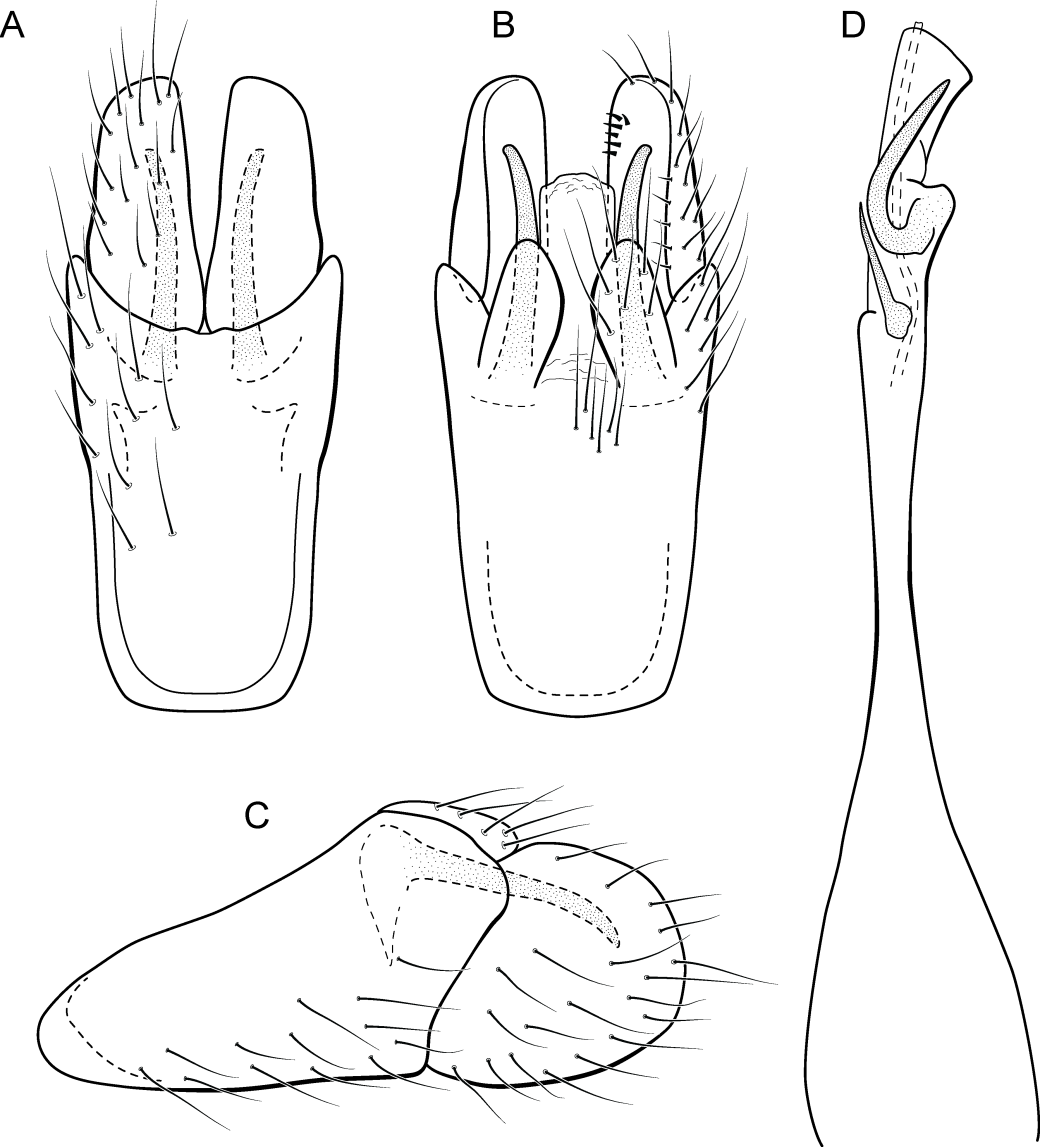
*Metrichia azul* **sp. nov.**, male genitalia. (A) ventral view; (B) dorsal view; (C) lateral view; (D) phallus, dorsal view.

**Adult male.** Length 2.0–2.1 mm (*n* = 4). General color, in alcohol, brown. Head with no modifications. Ocelli 3. Maxillary palpus 5-articulated, article IV broad and darkened; labial palpus 3-articulated. Mesoscutellum with transverse suture. Metascutellum subtriangular. Anterior femur without processes. Tibial spur formula 1-3-4. Wing venation reduced in both wings. Abdominal segment V with pair of internal pouches; segment VI with pair of internal pouches and pair of lateral external sacs with specialized setae. Ventromesal process on segment VII present. Segment VIII shorter ventrally than dorsally. **Male genitalia**. Segment IX reduced dorsally; sternum subrectangular (Fig. 6A); in lateral view narrower anteriorly than posteriorly (Fig. 6C). Inferior appendage covered by long setae and with scale-like setae; subrectangular in ventral view (Fig. 6A); in lateral view, rounded, apex rounded (Fig. 6C). Dorsal hook long, more than half length of inferior appendage; in lateral view, slightly downturned (Fig. 6C). Preanal appendage elongate, but shorter than inferior appendage, and bearing very long setae (Fig. 6B). Subgenital plate apparently absent. Tergum X membranous and subrectangular (Fig. 6B). Phallus tubular, elongate and slender, slightly constricted mesally, with two long, curved, subapical spines; apex rounded and sclerotized; ejaculatory duct sclerotized, sinuous, and protruding apically (Fig. 6D).

**Holotype.** **BRAZIL: Paraná:** Céu Azul, Parque Nacional do Iguaçu, Rio Azul, $25\textdegree 0{9}^{^{\prime}}2{1}^{^{\prime}^{\prime}}\mathrm{S}$ $53\textdegree 4{7}^{^{\prime}}4{4}^{^{\prime}^{\prime}}\mathrm{W}$, el. 510 m, 6–8 ix.2012, APM Santos, DM Takiya, ALH Oliveira, GA Jardim & BHL Sampaio cols., Malaise trap, male (DZRJ).

**Paratypes.** Same data as holotype, 2 males (DZRJ), 1 male (MNRJ).

**Etymology.** The specific name refers to the type locality, Rio Azul in the municipality of Céu Azul.

**Remarks**. This new species is another member of the *penicillata* group based on internal pouches between segment V and VI and the long subapical spines of the phallus. The new species shares similarities of the male genitalia with *M*. *biungulata* (Flint Jr, 1972) and *M*. *decora* Bueno-Soria & Holzenthal, 2003 particularly the rounded aspect of inferior appendages, but can be easily distinguished from those species by the absence of tooth-like processes on inferior appendages; more elongate preanal appendages; and dorsal hook only slightly downturned in lateral view.

### *Metrichia bonita* sp. nov.

urn:lsid:zoobank.org:act:622BCD51-CC39-4D1F-BDCB-84A7DFF4F071

(Figs. 7 and 8)

**Figure 7:**
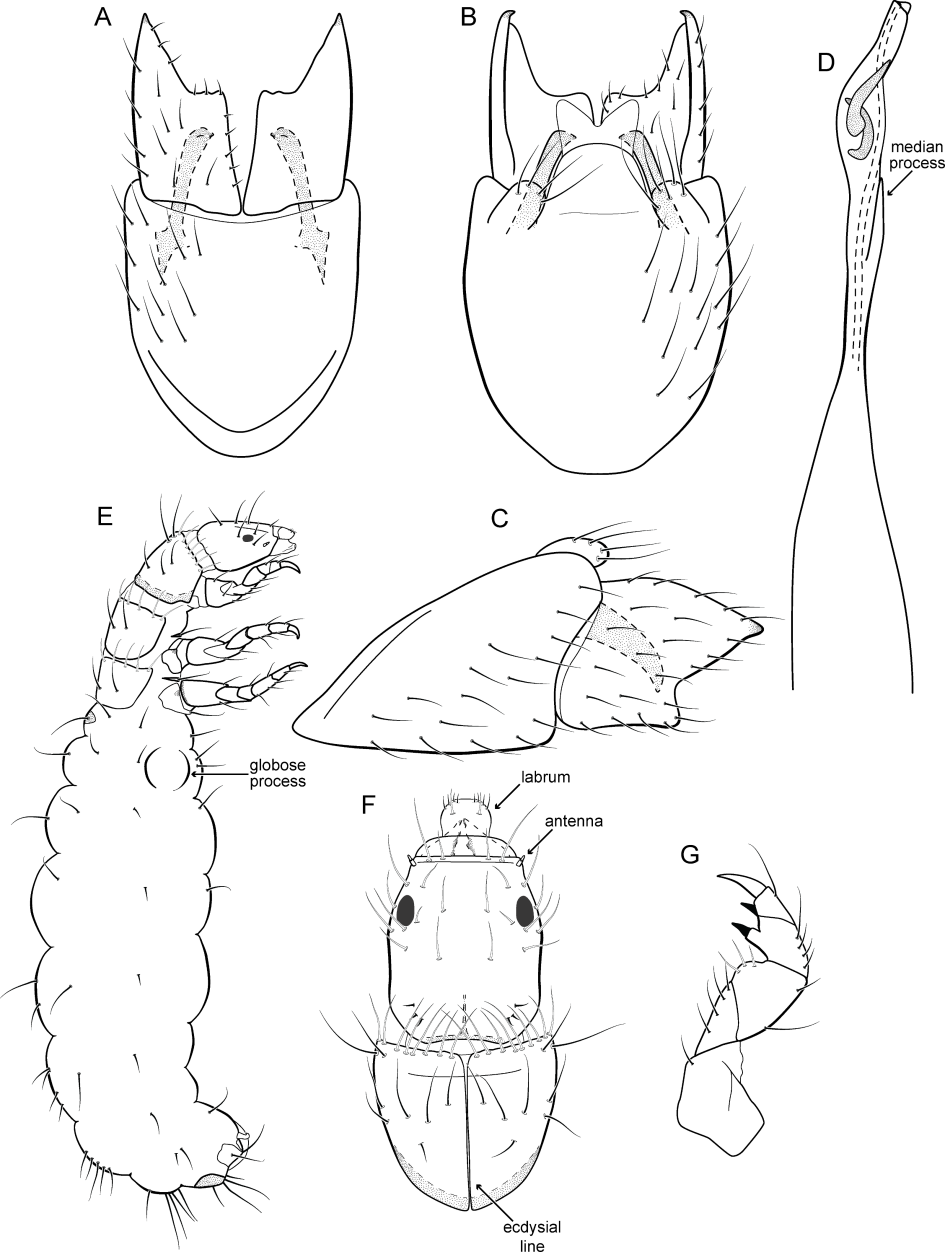
*Metrichia bonita* sp. nov. (A) male genitalia, ventral view; (B) male genitalia, dorsal view; (C) male genitalia, lateral view; (D) phallus, dorsal view; (E) larva, habitus, lateral view; (F) larva, head and pronotum, dorsal view; (G) larva, foreleg, ventral view.

**Figure 8:**
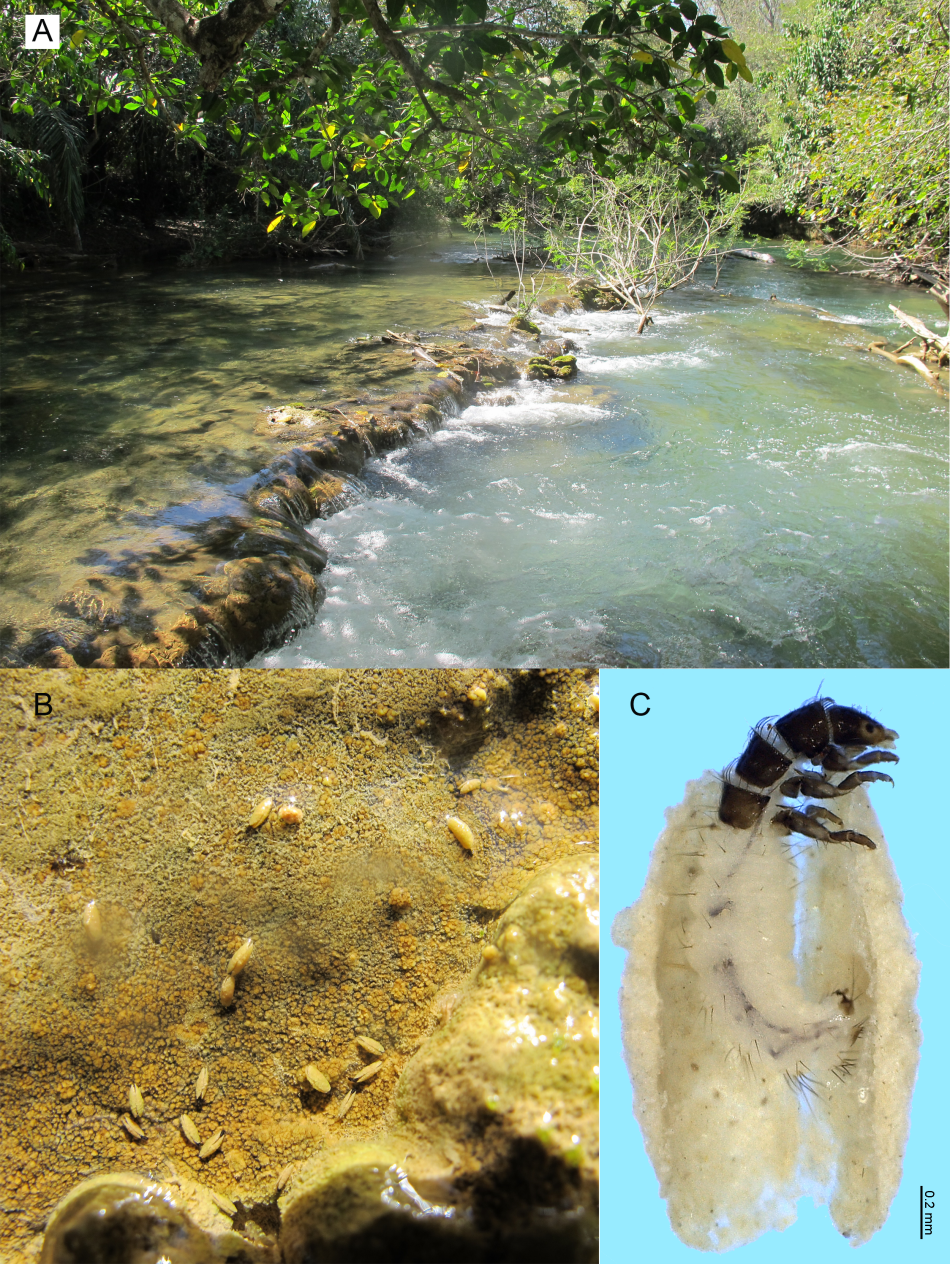
*Metrichia bonita* **sp. nov.**, larva. (A) type locality, Rio Formosinho, Bonito municipality, Mato Grosso do Sul, Brazil; (B) larvae on calcareous substrate; (C) larva and its calcareous case.

**Adult male.** Length 2.3–2.5 mm (*n* = 4). General color, in alcohol, brown. Head with no modifications. Ocelli 3. Antenna simple, 21-articulated. Maxillary palpus 5-articulated; labial palpus 3-articulated. Mesoscutellum with transverse suture. Metascutellum subtriangular. Anterior femur without processes. Tibial spur formula 1-3-4. Wing venation reduced in both wings. Abdominal segment VI with dorsal pouches covered with setae directed inward. Ventromesal process on segment VII absent. Segment VIII shorter ventrally than dorsally. **Male genitalia**. Segment IX reduced dorsally; sternum subrectangular, with anterior margin rounded (Fig. 7A); in lateral view narrower anteriorly than posteriorly (Fig. 7C). Inferior appendage covered by long setae, subtrapezoidal in ventral view (Fig. 7A); in lateral view, with an acute projection, apex with acute corners (Fig. 7C). Dorsal hook short, almost half length of inferior appendage; in lateral view, slightly downturned (Fig. 7C). Preanal appendage short, rounded and bearing very long setae (Fig. 7B). Subgenital plate apparently absent. Tergum X membranous and with a shallow U-shaped incision (Fig. 7B). Phallus tubular, elongate and slender, slightly constricted mesally, with a median process; with two curved subapical spines, one short and another long; apex truncate and sclerotized; ejaculatory duct sclerotized and not protruding apically (Fig. 7D).

**Larva (5th instar).** Length 1.5–1.9 mm (*n* = 10). Head dark brown, unpigmented around stemmata (Fig. 8C); slightly longer than broad; frontoclypeal and coronal sulci indistinct; with a few long setae (Fig. 7F). Antenna short, apparently 2-articulated and with no apical setae (Fig. 7F). Labrum with pair of stout setae (Fig. 7F). Mandibles with inner margin sinuous and darkened. Thoracic nota sclerotized, dark brown, with a row of stout setae on anterior margin. Pro-, meso-, and metanotum with middorsal ecdysial line. Thoracic segments with small pleurites (Fig. 7E). Thoracic legs brown, short and stout, almost the same size of each other. Foreleg with stout setae; tibia with a posteroventral lobe with a spine-like seta (Fig. 7G); femur bearing a spine-like setae; tarsal claw simple. Mid- and hind legs with stout setae on posteroventral margin. Abdomen almost white, with dark brown sclerites. Abdominal segment I with ellipsoid tergite; segments I–V with pair of long, dorsal setae and pair of dorsolateral setae; segment VI with two pairs of long dorsal setae and two pairs of dorsolateral setae; segments VII and VIII with three pairs of dorsal, long setae and two pair of dorsolateral setae; segment IX with sclerotized tergite and several long setae. Abdominal segments I, III, IV, and IX with pair of ventral, long setae; segment II with two pairs of ventral, long setae and globose process on ventrolateral area (Fig. 7E). Anal proleg very short not projecting prominently; with basal sclerite bearing long setae; anal claw simple.

**Larval case.** Length 1.5–2.0 mm (*n* = 10). General color white (Figs. 8B and 8C). Constructed with calcareous particles (with no algal filaments added), forming two rigid and lateral valves, poorly closed dorsally and ventrally (Fig. 8C). External surface rugose.

**Biology.** Larvae were collected on calcareous tufa in a fast flowing river, approximately 10 m wide (Fig. 8A). No pupae were found and adults were not seen active during the day.

**Holotype. BRAZIL: Mato Grosso do Sul:** Bonito, Rio Formosinho, $21\textdegree 1{0}^{^{\prime}}1{6}^{^{\prime}^{\prime}}\mathrm{S}$ $56\textdegree 2{6}^{^{\prime}}4{7}^{^{\prime}^{\prime}}\mathrm{W}$ el. 275 m, 08–13.ix.2013, APM Santos & DM Takiya cols., Malaise trap, male (DZRJ).

**Paratypes.** Same data as holotype, 3 males (DZRJ), 1 male (MNRJ).

**Additional material.** Same data as holotype, except 13.ix.2013, manual, 10 larvae (DZRJ), 10 larvae (MNRJ).

**Etymology.** This species is named in reference to the type locality (Fig. 8), the municipality of Bonito in the state of Mato Grosso do Sul. In Portuguese, the word “bonita” (the feminine form) means “beautiful”.

**Remarks**. *Metrichia bonita* **sp. nov.** has features of the *nigritta* group: internal pouches between segment V–VI and phallus with 2 spines and an acute process on distal portion. This new species can be easily distinguished from other species in this group based on the shape of inferior appendages, subtrapezoidal in ventral view, with dorsal corners acute and darkened. In addition, inferior appendages have dorsal hooks, which are broad basally and slightly downturned in lateral view. COI sequences showed maximum intraspecific distance of 1.5% and minimum interspecific distance of 21.0% to its closest neighbor, *M*. *itabaiana* **sp. nov.** Although the male genitalia of both species show some superficial resemblance, based on the abdominal modifications and phallic aspect, *M. bonita* **sp. nov.** belongs to the *nigritta* group, whereas *M*. *itabaiana* fits better in the *campana* group.

Larvae of *Metrichia bonita* **sp. nov.** are very similar to those previously described or illustrated, including *M*. *nigritta*, *M*. *juana*, and unassociated larvae illustrated by Botosaneanu & Flint Jr (1982) from Venezuela and Ecuador; and by Pes, Hamada & Nessimian (2005) from Brazil. Actually, main differences seem to be the shape and the material of larval cases. In this respect, larvae of *Metrichia bonita* **sp. nov.** are unusual and easily recognized by having their case made entirely of calcareous particles, without typical algal elements (Fig. 8).

Paprocki, Holzenthal & Cressa (2003) discussed the role of *Smicridea travertinera* Paprocki, Holzenthal & Cressa, 2003 in calcareous tufa formation (travertine). According to these authors, larvae of that species interfere in the deposition and erosion of the calcareous substrate by their net-building activities (Paprocki, Holzenthal & Cressa, 2003). Cyanobacteria and diatoms are known to participate in travertine formation, but the role played by macroinvertebrates is poorly understood (Drysdale, 1998; Drysdale, 1999). It is possible that cases of *Metrichia bonita* **sp. nov.** are impregnated passively with calcareous particles, but as commented by Drysdale (1999) for other aquatic insects, they could be important in travertine biogenesis by producing new nucleation sites or eroding other ones. *Metrichia bonita* **sp. nov.** is the only microcaddisfly known to inhabit (Fig. 8) and build cases with calcareous tufa so far.

### *Metrichia bracui* sp. nov.

urn:lsid:zoobank.org:act:07B44840-CAB0-4BBE-BD01-B62E885BE418

(Fig. 9)

**Figure 9:**
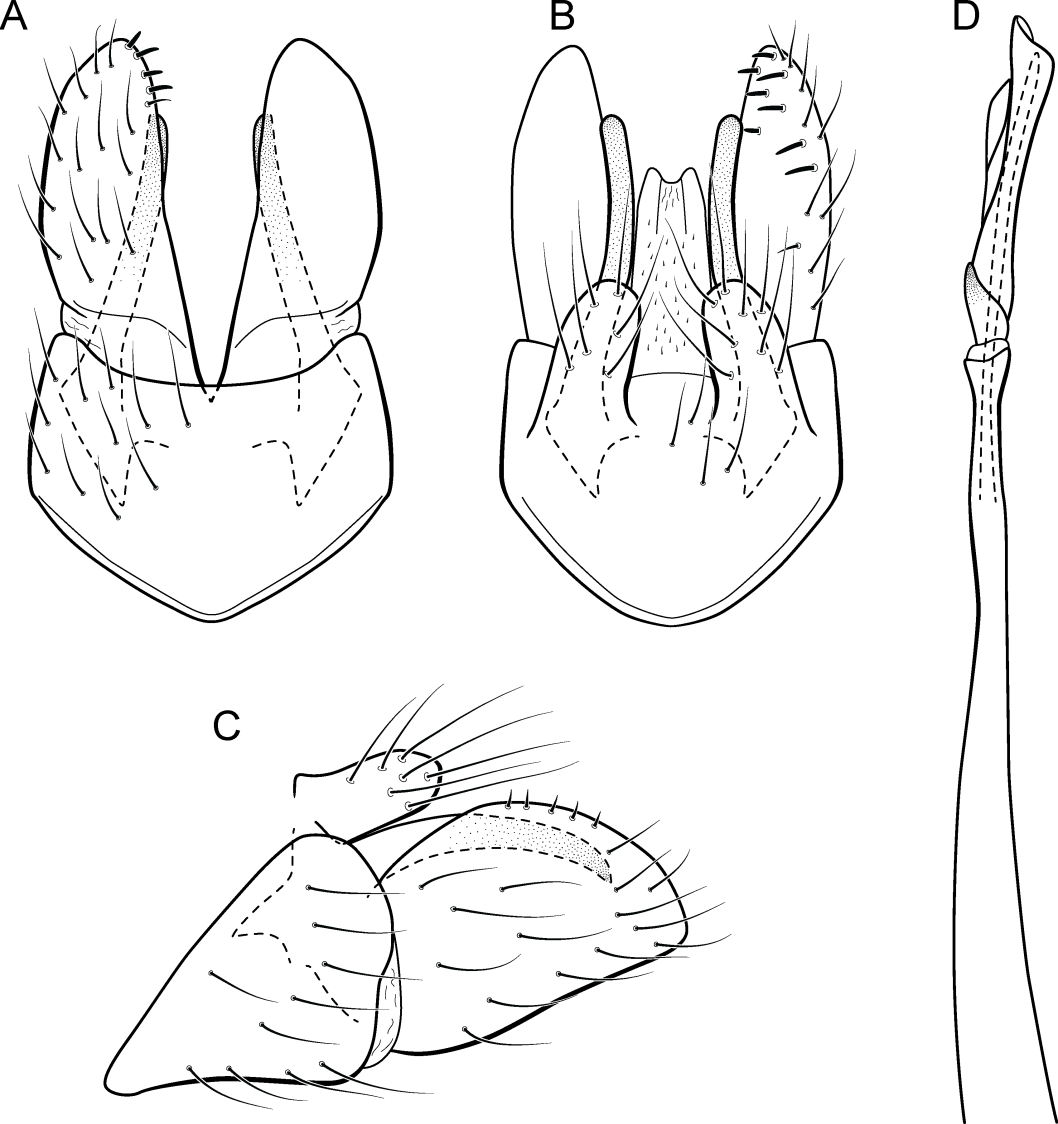
*Metrichia bracui* **sp. nov.**, male genitalia. (A) ventral view; (B) dorsal view; (C) lateral view; (D) phallus, dorsal view.

**Adult male.** Length 1.8–2.2 mm (*n* = 3). General color, in alcohol, light brown. Head with no modifications. Ocelli 3. Antenna simple, 18-articulated. Maxillary palpus 5-articulated; labial palpus 3-articulated. Mesoscutellum with transverse suture. Metascutellum subtriangular. Anterior femur without processes. Tibial spur formula 1-3-4. Wing venation reduced in both wings. Abdomen without modifications. Ventromesal process on segment VII absent. Segment VIII shorter ventrally than dorsally. **Male genitalia**. Segment IX reduced dorsally; sternum subpentagonal in ventral view (Fig. 9A); in lateral view narrower anteriorly than posteriorly (Fig. 9C). Inferior appendage covered by long setae and with scale-like setae, subrectangular in ventral view (Fig. 9A); in lateral view, rounded, apex rounded (Fig. 9C). Dorsal hook long, more than half length of inferior appendage; in lateral view, downturned (Fig. 9C). Preanal appendage elongate and bearing very long setae (Fig. 9B). Subgenital plate apparently absent. Tergum X membranous and with shallow U-shaped incision (Fig. 9B). Phallus tubular, elongate and slender, slightly constricted mesally; without spines, but with a sclerotized process arising from a subapical constriction; apex rounded and folded; ejaculatory duct sclerotized, straight and not protruding apically (Fig. 9D).

**Holotype.** **BRAZIL: Rio de Janeiro:** Angra dos Reis, Rio Bracuí, $23\textdegree 0{0}^{^{\prime}}2{3}^{^{\prime}^{\prime}}\mathrm{S}$ $44\textdegree 2{9}^{^{\prime}}1{5}^{^{\prime}^{\prime}}\mathrm{W}$, el. 75 m, 10–11.v.2002, JL Nessimian col., light trap, male (DZRJ).

**Paratypes.** Same data as holotype, 2 males (MNRJ). **Rio de Janeiro:** Parque Nacional do Itatiaia, Córrego do Maromba, $22\textdegree 2{5}^{^{\prime}}3{2}^{^{\prime}^{\prime}}\mathrm{S}$ $44\textdegree 3{7}^{^{\prime}}0{3}^{^{\prime}^{\prime}}\mathrm{W}$, el. 1,250 m, 04.iv.15, APM Santos & DM Takiya cols., Malaise trap, 4 males (DZRJ).

**Etymology.** The species is named in allusion to the river where the holotype was collected.

**Remarks**. This new species can be assigned to the *patagonica* group because of the absence of curved spines of the phallus. The general aspect of the male genitalia resembles *M. patagonica* (Flint, 1983), *M*. *pernambucana*, and *M*. *pseudopatagonica* Bueno-Soria & Holzenthal, 2003. *Metrichia bracui* **sp. nov.** differs from these species and others in the group specially by the phallus bearing a sclerotized process on a constricted region.

The four COI sequences generated for *M*. *bracui* **sp. nov.** were from specimens collected in a locality at Parque Nacional do Itatiaia, Rio de Janeiro, Brazil. The highest pairwise intraspecific divergence between sequences was 0.4%, and the lowest interspecific divergence was 21.4% between *M*. *bracui* **sp. nov.** and *Angrisanoia cebollati* (Angrisano, 1995). Until now, there have been no formal studies on the relationships among species or genera included in Ochrotrichiinae. However, to infer phylogenetic hypothesis for the entire subfamily is beyond the scope of this work, and also much more data and taxa sampling are necessary to generate robust hypotheses.

### *Metrichia caraca* sp. nov.

urn:lsid:zoobank.org:act:34F912BC-1069-433E-AF16-A1B19D7FA622

(Fig. 10)

**Figure 10:**
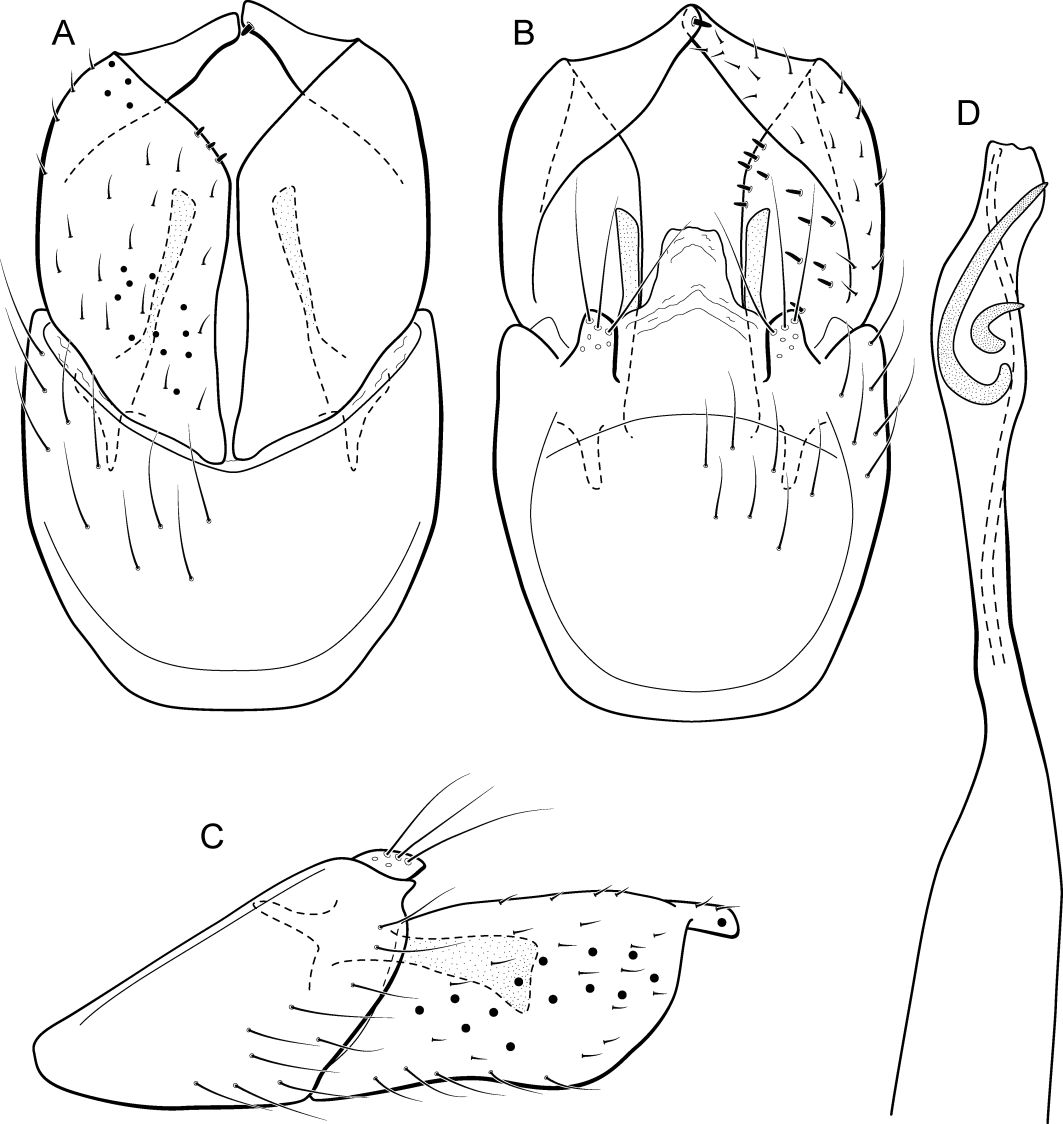
*Metrichia caraca* **sp. nov.**, male genitalia. (A) ventral view; (B) dorsal view; (C) lateral view; (D) phallus, dorsal view.

**Adult male.** Length 2.5–3.0 mm (*n* = 5). General color, in alcohol, brown. Head with no modifications. Ocelli 3. Antenna simple, 18-articulated. Maxillary palpus 5-articulated; labial palpus 3-articulated. Mesoscutellum with transverse suture. Metascutellum subtriangular. Anterior femur without processes. Tibial spur formula 1-3-4. Wing venation reduced in both wings. Abdominal segment VI bearing brush of very long setae dorsolateraly; segment VII bearing brush of very long setae dorsolateraly. Ventromesal process on segment VII absent. Segment VIII shorter ventrally than dorsally and bearing brush of long setae dorsally. **Male genitalia**. Segment IX reduced dorsally; sternum subrectangular, with anterior margin rounded (Fig. 10A); in lateral view narrower anteriorly than posteriorly (Fig. 10C). Inferior appendage covered by long setae, subtrapezoidal in ventral view (Fig. 10A), apex oblique and projected mesad into a large process bearing a stout spine-like setae. Dorsal hook short and straight; in lateral view, truncate and broader apically (Fig. 10C). Preanal appendage short, truncate and bearing very long setae (Fig. 10B). Subgenital plate apparently absent. Tergum X membranous and truncate (Fig. 10B). Phallus tubular, elongate and slender, slightly constricted mesally; with two curved subapical spines, one short and another long; apex emarginate; ejaculatory duct sclerotized and not protruding apically (Fig. 10D).

**Holotype.** **BRAZIL: Minas Gerais:** Catas Altas, RPPN Santuário do Caraça, Ribeirão Caraça, 11–13.vi.2013, ML Monné & JP Botero cols., Malaise trap, male (DZRJ).

**Paratypes.** Same data as holotype, 3 males (DZRJ). **Minas Gerais:** São Roque de Minas, Parque Nacional da Serra da Canastra, Fazenda Velha, Córrego dos Pombos, $20\textdegree 1{4}^{^{\prime}}5{7}^{^{\prime}^{\prime}}\mathrm{S}$ $46\textdegree 3{8}^{^{\prime}}0{5}^{^{\prime}^{\prime}}\mathrm{W}$, el. 997 m, 02.iv.2014, JL Nessimian, ALH Oliveira, LL Dumas & SP Gomes, light trap cols., 1 male (MNRJ).

**Etymology.** This species is named in reference to the stream where type specimens were collected.

**Remarks**. This new species has very distinctive male genitalia. Based on the dorsoapically produced inferior appendages, it resembles *M. lenophora* (Flint, 1991). However, *M. caraca* **sp. nov.** is easily recognized by the obliquely truncate and mesad directed process of inferior appendages and dorsal hook, in lateral view, with apex broad and truncate.

COI distances within this species reached only 1.1% and the lowest interspecific distance (21.7%) was found between specimens of *M. caraca* **sp. nov.** and *M. acuminata* **sp. nov.**, which are very distinct based on morphological features.

### *Metrichia circuliforme* sp. nov.

urn:lsid:zoobank.org:act:E539EF55-F963-433C-BB25-C9177339ED54

(Fig. 11)

**Figure 11:**
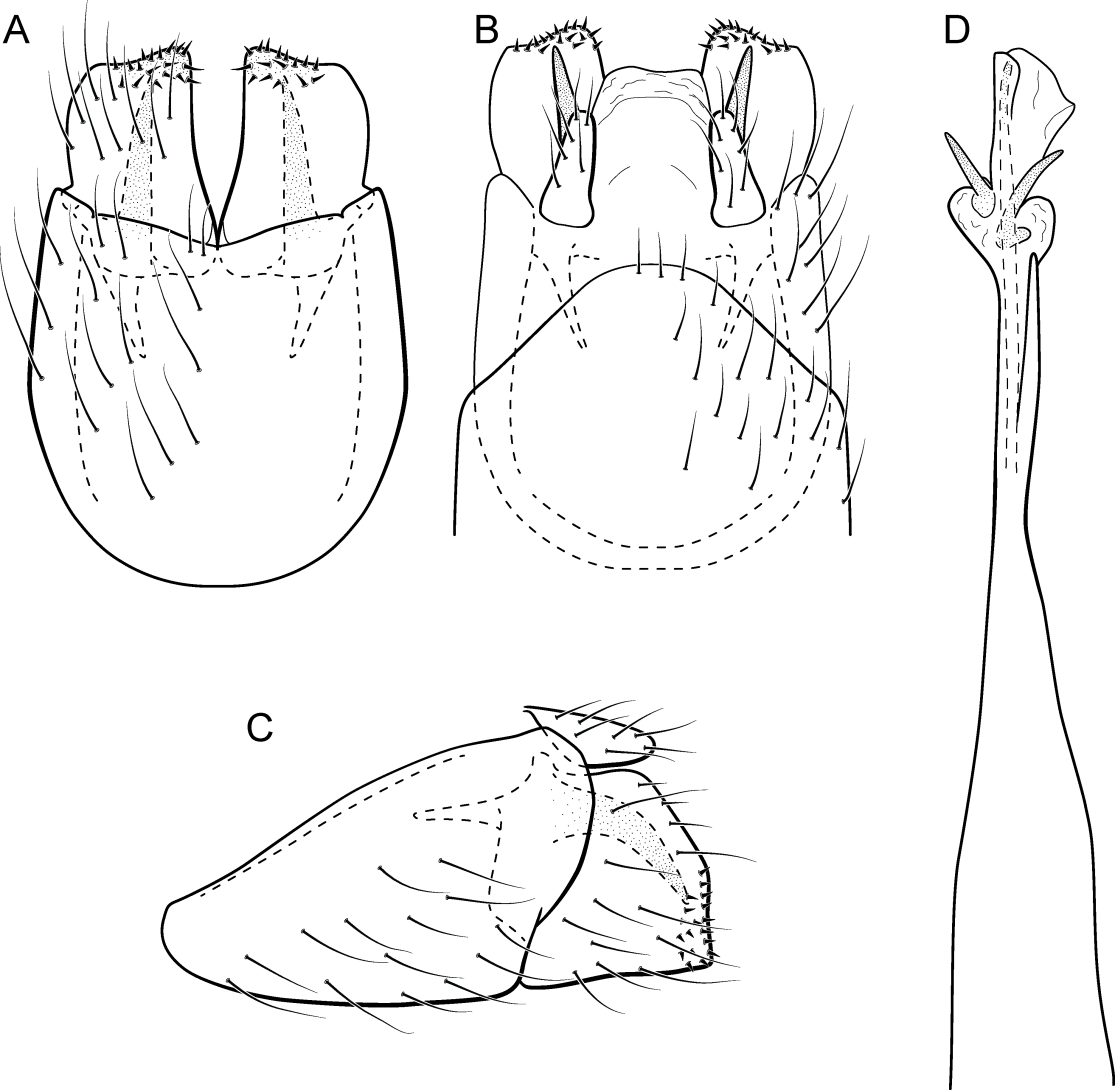
*Metrichia circuliforme* **sp. nov.**, male genitalia. (A) ventral view; (B) dorsal view; (C) lateral view; (D) phallus, dorsal view.

**Adult male.** Length 2.5–2.7 mm (*n* = 4). General color, in alcohol, brown. Head with no modifications. Ocelli 3. Antenna simple, 18-articulated. Maxillary palpus 5-articulated; labial palpus 3-articulated. Mesoscutellum with transverse suture. Metascutellum subtriangular. Anterior femur without processes. Tibial spur formula 1-3-4. Wing venation reduced in both wings. Abdominal segment VI bearing brush of very long setae dorsolateraly; segment VII bearing a brush of very long setae dorsolateraly. Ventromesal process on segment VII present. Segment VIII shorter ventrally than dorsally. **Male genitalia**. Segment IX reduced dorsally; sternum subpentagonal (Fig. 11A); in lateral view narrower anteriorly than posteriorly (Fig. 11C). Inferior appendage short, covered by long setae, subrectangular in ventral view (Fig. 11A); in lateral view, rounded (Fig. 11C), apex slightly truncate and bearing short spine-like setae. Dorsal hook long, almost reaching the inferior appendage apex; in lateral view, downturned (Fig. 11C). Preanal appendage elongate, but shorter than inferior appendage, and bearing very long setae (Fig. 11B). Subgenital plate apparently absent. Tergum X membranous and truncate (Fig. 11B). Phallus tubular, elongate and slender, slightly constricted mesally and with a median process; with two short subapical spines; apex rounded and sclerotized; ejaculatory duct sclerotized, straight and not protruding apically (Fig. 11D).

**Holotype.** **BRAZIL: Rio de Janeiro:** Itatiaia, Rio das Pedras, Cachoeira de Deus, $22\textdegree 2{5}^{^{\prime}}0{0}^{^{\prime}^{\prime}}\mathrm{S}$ $44\textdegree 3{2}^{^{\prime}}5{0}^{^{\prime}^{\prime}}\mathrm{W}$, el. 689 m, 06.iii.2008, JL Nessimian, LL Dumas & MR de Souza cols., light trap, male (DZRJ).

**Paratypes.** Same data as holotype, except Rio das Pedras, $22\textdegree 2{4}^{^{\prime}}3{3}^{^{\prime}^{\prime}}\mathrm{S}$ $44\textdegree 3{3}^{^{\prime}}0{8}^{^{\prime}^{\prime}}\mathrm{W}$, el. 706 m, 06.iii.2008, LL Dumas, JL Nessimian & MR de Souza cols., light trap, 1 male (DZRJ), 1 male (MNRJ); Parque Nacional do Itatiaia, Córrego Simon, $22\textdegree 2{6}^{^{\prime}}1{6}^{^{\prime}^{\prime}}\mathrm{S}$ $44\textdegree 3{6}^{^{\prime}}2{0}^{^{\prime}^{\prime}}\mathrm{W}$, el. 1,033 m, 15.iv.07, LL Dumas, APM Santos, N Ferreira-Jr. & JL Nessimian cols., light trap, 1 male (DZRJ).

**Etymology.** The new species name is an allusion to the rounded and simple inferior appendages, derived from the Latin, “circuli-” and “form” meaning “rounded shape”.

**Remarks**. This species has simple male genitalia and abdomen with only brushes of long setae on segments VI and VII. General aspect of the male genitalia is similar to *M*. *riva* (Bueno-Soria, 1983) and *M*. *quadrata* (Flint Jr, 1972), particularly their inferior appendages short and subrectangular and phallus with two subapical spines. However, *M*. *circuliforme* **sp. nov.** can be easily distinguished from *M*. *quadrata* by the absence of internal sacs in the abdomen. It can be distinguished from *M*. *riva* by the elongate preanal appendages and phallus with subequal hook spines subapically.

We obtained seven COI sequences for *M*. *circuliforme* **sp. nov.** and although all of them came from specimens collected at the same locality, intraspecific divergences were relatively high, reaching 3.5%. Besides that, GMYC estimated two species for these sequences instead of one. Re-analysis of the morphology of these specimens did not reveal any conspicuous variation that could justify splitting this species into two taxonomic groups. Compared to other studies using DNA barcodes of caddisflies, this genetic distance is still low, for example Pauls et al. (2010) found intraspecific divergences (K2P distance) up to 5.9% for Chilean *Smicridea*. Zhou et al. (2011) found even higher intraspecific distances among caddisflies, reaching up to 14%. GMYC is known to be more sensitive to geographic range coverage and/or other sampling schemes, resulting in oversplitting (Lohse, 2009; Talavera, Dincă & Vila, 2013). Therefore, we consider *M*. *circuliforme* **sp. nov.** as a robust species based on morphology as well as based on barcode divergences. Minimum interspecific COI distances of *M. circuliforme* **sp. nov.** to *M*. *curta* **sp. nov.** were 18.4%, and again, these two species are very distinct based on morphological features and apparently are not even closely related to each other (Fig. 2).

### *Metrichia curta* sp. nov.

urn:lsid:zoobank.org:act:7EC0620B-D6F2-409C-8351-79A3D3FB77C5

(Fig. 12)

**Figure 12:**
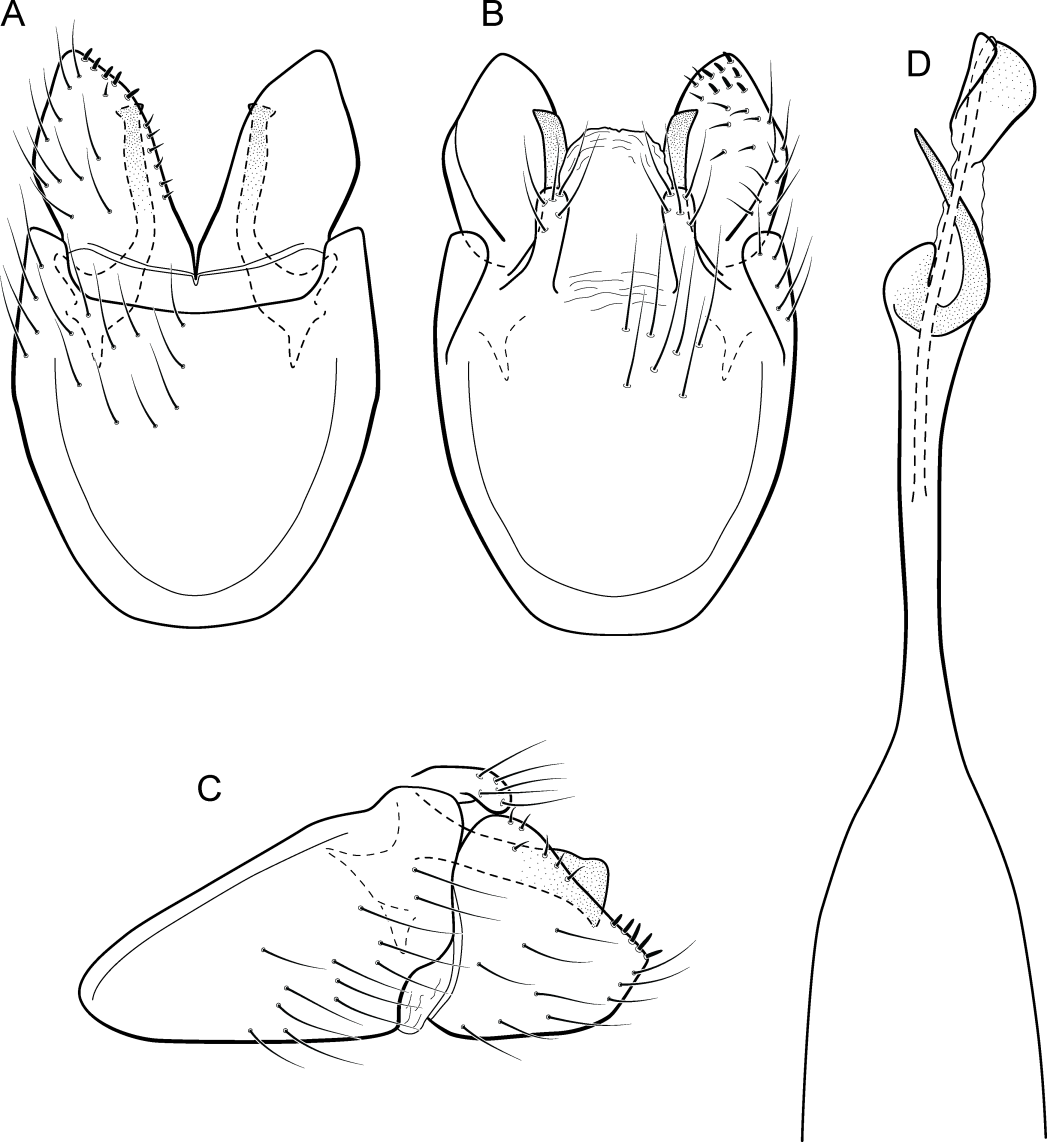
*Metrichia curta* **sp. nov.**, male genitalia. (A) ventral view; (B) dorsal view; (C) lateral view; (D) phallus, dorsal view.

**Adult male.** Length 2.4–2.5 mm (*n* = 7). General color, in alcohol, light brown. Head with no modifications. Ocelli 3. Antenna simple, 18-articulated. Maxillary palpus 5-articulated; labial palpus 3-articulated. Mesoscutellum with transverse suture. Metascutellum subtriangular. Anterior femur without processes. Tibial spur formula 1-3-4. Wing venation reduced in both wings. Abdomen without modifications. Ventromesal process on segment VII present. Segment VIII shorter ventrally than dorsally. **Male genitalia**. Segment IX reduced dorsally; sternum subpentagonal (Fig. 12A); in lateral view, narrower anteriorly than posteriorly (Fig. 12C). Inferior appendage covered by long setae, subtrapezoidal in ventral view (Fig. 12A); in lateral view, subtriangular (Fig. 12C), apex rounded. Dorsal hook long, more than half length of inferior appendage; in lateral view, with apex slightly broader, downturned, and truncate (Fig. 12C). Preanal appendage elongate, but shorter than inferior appendage, and bearing very long setae (Fig. 12B). Subgenital plate apparently absent. Tergum X membranous and rounded (Fig. 12B). Phallus tubular, elongate and slender, slightly constricted mesally; with a stout subapical spine; apex rounded and folded; ejaculatory duct sclerotized, straight and protruding apically (Fig. 12D).

**Holotype.** **BRAZIL: Rio de Janeiro:** Itatiaia, Rio das Pedras, $22\textdegree 2{4}^{^{\prime}}3{3}^{^{\prime}^{\prime}}\mathrm{S}$ $44\textdegree 3{3}^{^{\prime}}0{8}^{^{\prime}^{\prime}}\mathrm{W}$, el. 706 m, 06.iii.2008, LL Dumas, JL Nessimian & MR de Souza cols., light trap, male (DZRJ).

**Paratypes.** Same data as holotype, 3 males (DZRJ), 3 males (MNRJ).

**Etymology.** The specific name is a reference to the very short inferior appendage; in Portuguese “curta” means “short.”

**Remarks**. Based on the absence of modifications on abdominal segments, this new species can be assigned to the *aberrans* group. Its phallus is similar to that of *M*. *amplitudinis* Bueno-Soria & Holzenthal, 2003, with a lonjg spine and an apical flap. The new species can be distinguished by the triangular inferior appendages in lateral view and phallus with a strongly curved spine subapically. *Metrichia amplitudinis* and *M*. *curta* **sp. nov.** share the widened dorsal hook, but in the new species this structure is only slightly wider and also truncate in lateral view. Maximum intraspecific divergence of COI sequences was 1.5% for *M*. *curta* **sp. nov.** and minimum interspecific was to *M*. *circuliforme*, as mentioned above.

### *Metrichia farofa* sp. nov.

urn:lsid:zoobank.org:act:BC4FF095-32BE-46A9-BEAB-28854E2F5BC7

(Fig. 13)

**Figure 13:**
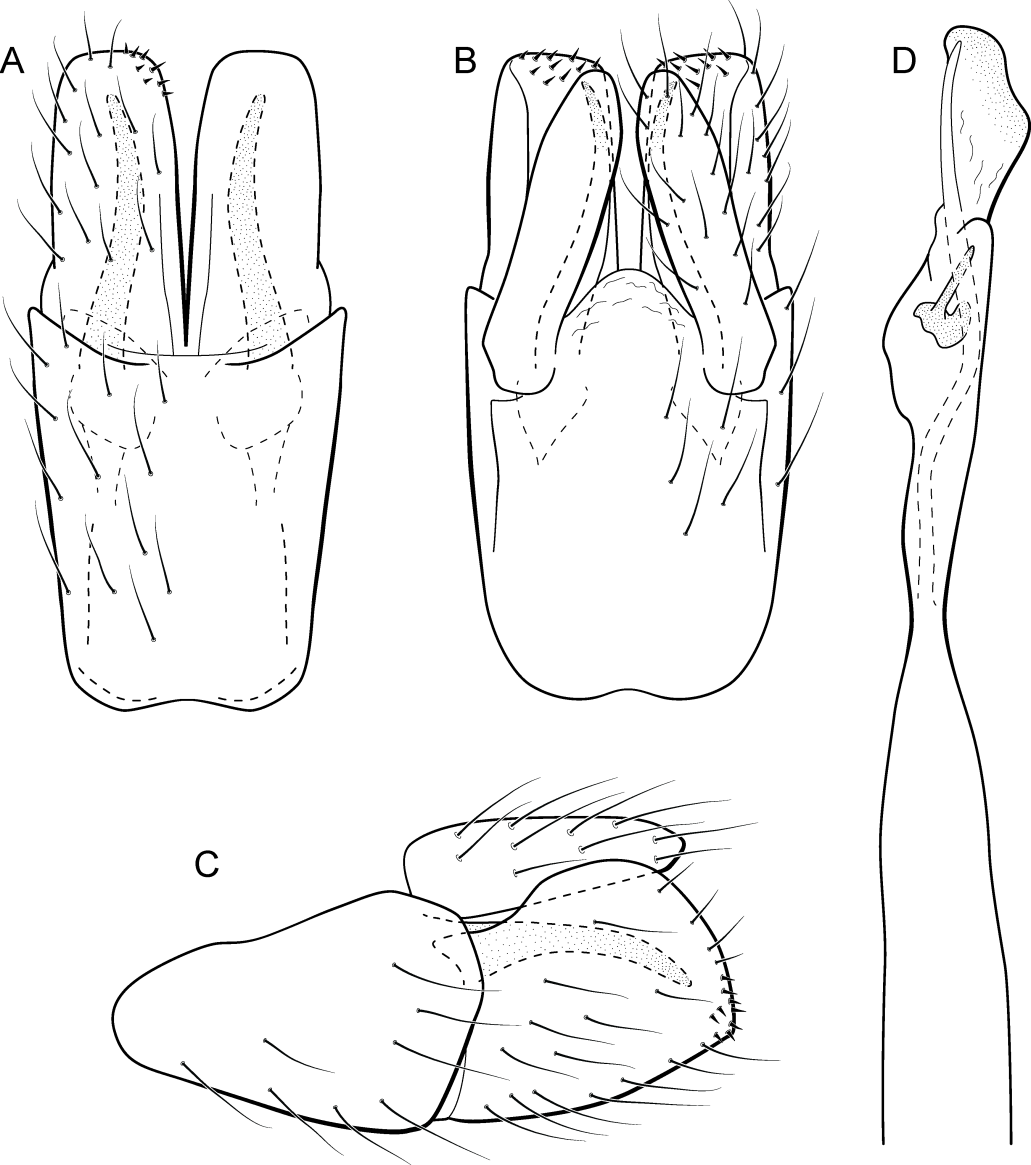
*Metrichia farofa* **sp. nov.**, male genitalia. (A) ventral view; (B) dorsal view; (C) lateral view; (D) phallus, dorsal view.

**Adult male**. Length 1.8–2.1 mm (*n* = 27). General color, in alcohol, light brown. Head with no modifications. Ocelli 3. Antenna simple, 18-articulated. Maxillary palpus 5-articulated; labial palpus 3-articulated. Mesoscutellum with transverse suture. Metascutellum subtriangular. Anterior femur without processes. Tibial spur formula 1-3-4. Wing venation reduced in both wings. Abdomen without modifications; segment VII bearing specialized setae dorsally. Ventromesal process on segment VII absent. Segment VIII shorter ventrally than dorsally. **Male genitalia**. Segment IX reduced dorsally; sternum subrectangular (Fig. 13A); in lateral view narrower anteriorly than posteriorly (Fig. 13C). Inferior appendage covered by long setae, subrectangular in ventral view (Fig. 13A); in lateral view, subrectangular (Fig. 13C), apex slightly truncate and bearing short spine-like setae. Dorsal hook long, almost reaching the inferior appendage apex; in lateral view, downturned (Fig. 13C). Preanal appendage elongate, as long as inferior appendage, and bearing very long setae (Fig. 13B). Subgenital plate apparently absent. Tergum X membranous and rounded (Fig. 13B). Phallus tubular, elongate and slender, slightly constricted mesally; with a stout subapical spine; apex rounded and sclerotized; ejaculatory duct sclerotized, sinuous, and protruding apically (Fig. 13D).

**Holotype.** **BRAZIL: Minas Gerais:** Jaboticatubas, Parque Nacional da Serra do Cipó, Cachoeira da Farofa, $19\textdegree 2{2}^{^{\prime}}4{7}^{^{\prime}^{\prime}}\mathrm{S}$ $43\textdegree 3{4}^{^{\prime}}3{6}^{^{\prime}^{\prime}}\mathrm{W}$, el. 811 m, 23.iv.2010, APM Santos & DM Takiya cols., manual, male (DZRJ).

**Paratypes.** Same data as holotype, 18 males (DZRJ), 8 males (MNRJ), 5 males (MZUFBA); same data, except Ribeirão Mascates, $19\textdegree 2{4}^{^{\prime}}0{2}^{^{\prime}^{\prime}}\mathrm{S}$ $43\textdegree 3{4}^{^{\prime}}3{5}^{^{\prime}^{\prime}}\mathrm{W}$, el. 820 m, 09–11.xii.2011, APM Santos & DM Takiya cols., manual, 84 males (DZRJ).

**Etymology.** This new species is named in reference to the waterfall where specimens were collected.

**Remarks**. Due to absence of pouches in abdominal segments, *Metrichia farofa* **sp. nov.** can also be included in the *aberrans* group. However, the new species has a single subapical spine in phallus, like those species included in the *exclamationis* group. The new species can be easily distinguished from all other *Metrichia* species by the very long preanal appendages, reaching apices of inferior appendages in dorsal and lateral views.

Although more than 10 specimens of *M*. *farofa* **sp. nov.** were submitted to DNA extraction and many attempts of COI amplification via PCR were conducted, we were not able to obtain sequences of this species, even using recent material collected after 2013.

### *Metrichia forceps* sp. nov.

urn:lsid:zoobank.org:act:8F25D006-D59B-4C83-8CDE-2398368917AD

(Fig. 14)

**Figure 14:**
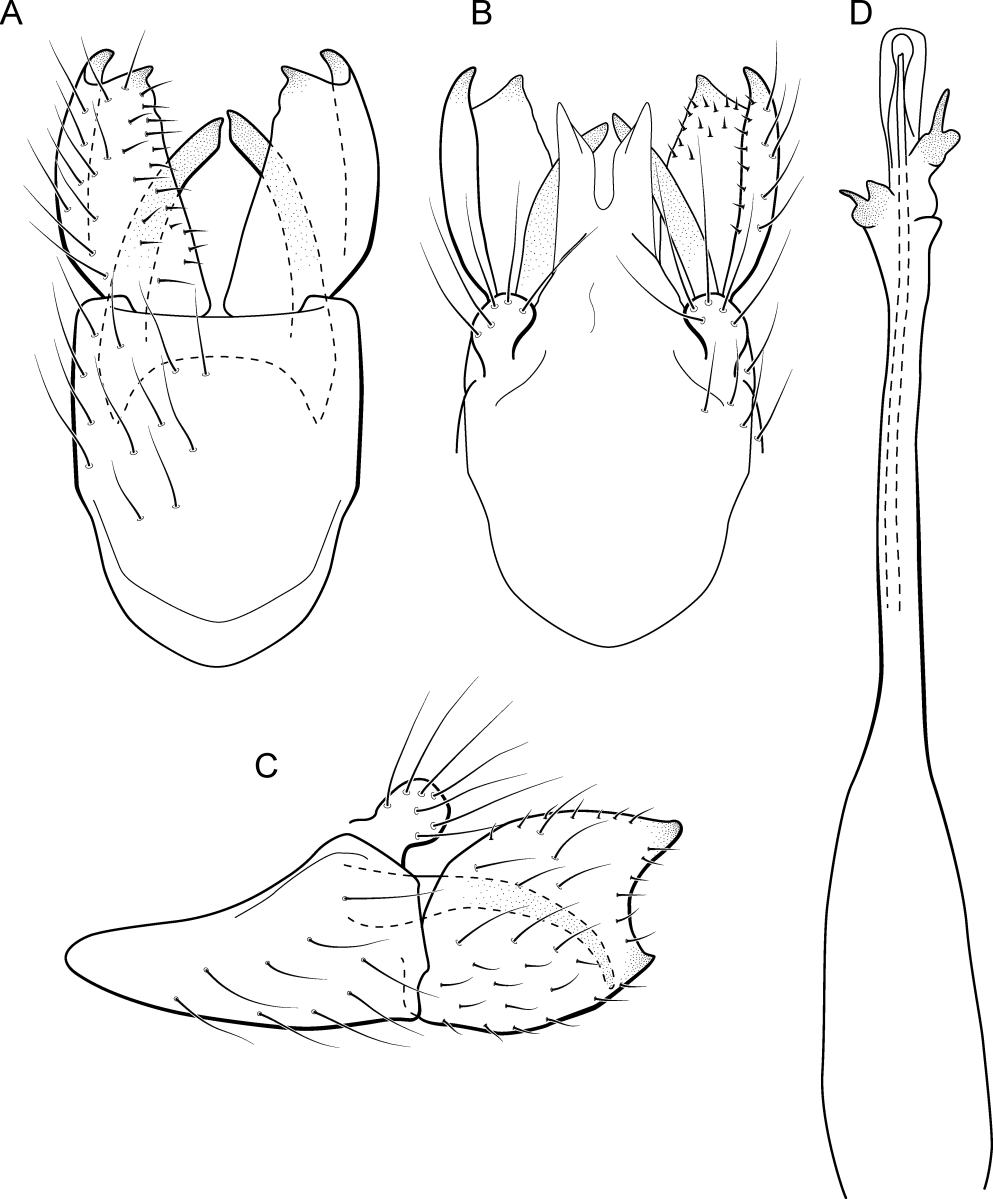
*Metrichia forceps* **sp. nov.**, male genitalia. (A) ventral view; (B) dorsal view; (C) lateral view; (D) phallus, dorsal view.

**Adult male**. Length 2.7–3.0 mm (*n* = 2). General color, in alcohol, brown. Head with no modifications. Ocelli 3. Antenna simple, 23-articulated. Maxillary palpus 5-articulated; labial palpus 3-articulated. Mesoscutellum with transverse suture. Metascutellum subtriangular. Anterior femur with small acute apical process. Tibial spur formula 1-3-4. Wing venation reduced in both wings. Abdominal segment VII bearing internal pouches in anterior area. Ventromesal process on segment VII absent. Segment VIII shorter ventrally than dorsally. **Male genitalia**. Segment IX reduced dorsally; sternum subpentagonal (Fig. 14A); in lateral view, narrower anteriorly than posteriorly (Fig. 14C). Inferior appendage covered by long setae, subrectangular in ventral view (Fig. 14A); in lateral view, with posterior margin excavated and with two acute and sclerotized process (Fig. 14C). Dorsal hook long, almost reaching the inferior appendage apex; in lateral view, downturned (Fig. 14C). Preanal appendage short, rounded and bearing very long setae (Fig. 14B). Subgenital plate apparently absent. Tergum X sclerotized, deeply notched mesally, forming lateral curved processes (Fig. 14B). Phallus tubular, elongate and slender, slightly constricted mesally; with two short subapical spines; apex rounded and folded; ejaculatory duct sclerotized, straight and protruding apically (Fig. 14D).

**Holotype.** **BRAZIL: Paraná:** Céu Azul, Parque Nacional do Iguaçu, Rio Azul, $25\textdegree 0{9}^{^{\prime}}2{1}^{^{\prime}^{\prime}}\mathrm{S}$ $53\textdegree 4{7}^{^{\prime}}4{4}^{^{\prime}}\mathrm{W}$, el. 510 m, 6–8 ix.2012, APM Santos, DM Takiya, ALH Oliveira, GA Jardim & BHL Sampaio cols., Malaise trap, male (DZRJ).

**Paratypes.** Same data as holotype, 1 male (MNRJ).

**Etymology.** The name of this species is in reference to the dorsal hooks of inferior appendages, which in ventral view resemble forceps.

**Remarks**. This new species belongs to the *campana* group, sharing the diagnostic internal pouches between segments VI and VII, reduced spines on subapical region of phallus, and the sclerotized and elongate tergum X. Within this group, *M*. *forceps* **sp. nov.** is most similar to *M*. *campana* (Flint Jr, 1968), *M*. *similis* (Flint Jr, 1968), and *M*. *continentalis* (Flint Jr, 1972), particularly by their inferior appendages with excavate posterior margins, forming two pointed processes, one ventral and another dorsal. The new species can be distinguished from the others by its deeply notched tergum X; dorsal hook of inferior appendages elongate and downturned, and phallus apex bearing two small spines and a sclerotized flap surrounding the protruding ejaculatory duct.

### *Metrichia formosinha* sp. nov.

urn:lsid:zoobank.org:act:B8971D9B-7013-4213-970E-50A42CA0D1B7

(Fig. 15)

**Figure 15:**
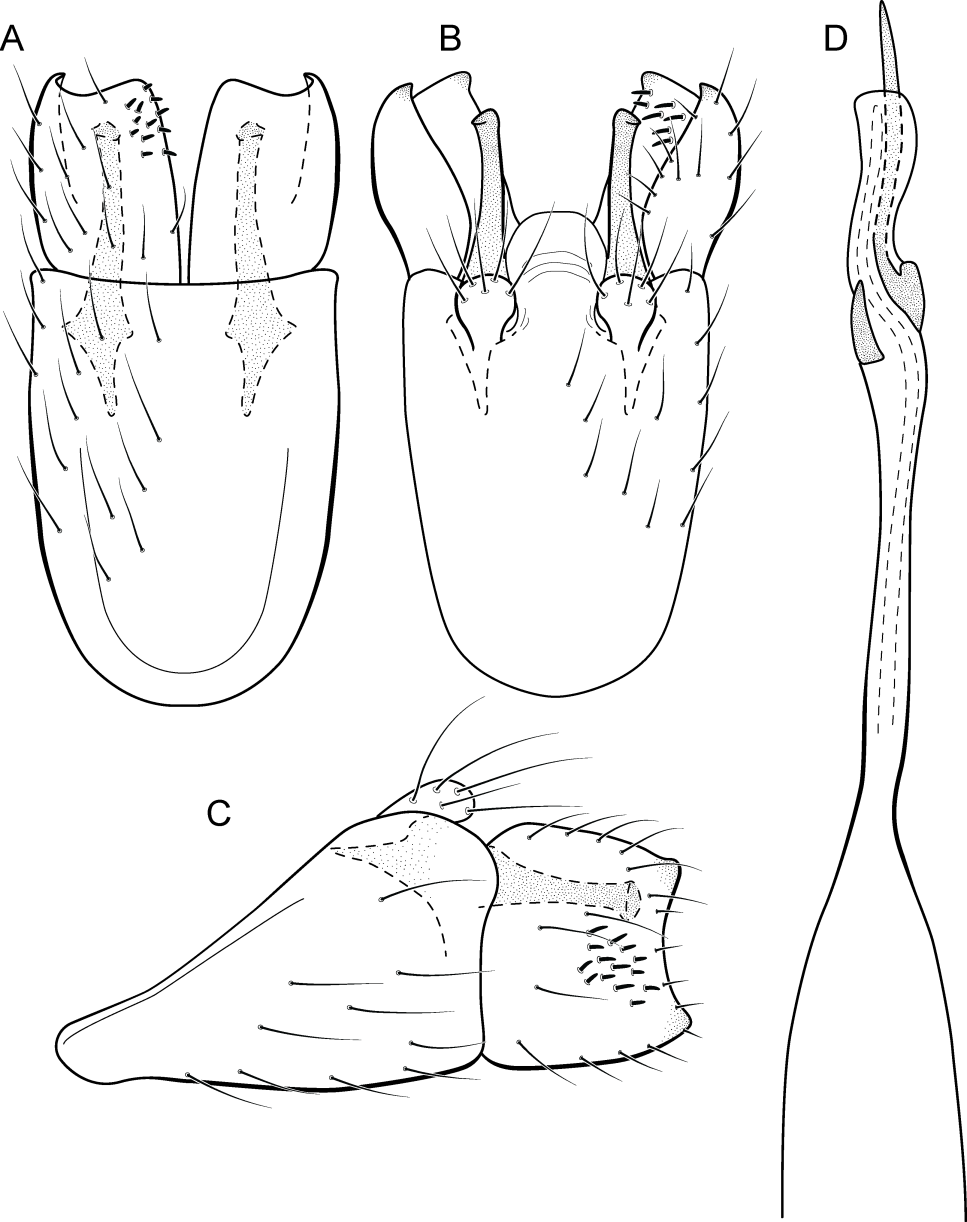
*Metrichia formosinha* **sp. nov.**, male genitalia. (A) ventral view; (B) dorsal view; (C) lateral view; (D) phallus, dorsal view.

**Adult male**. Length 2.5–2.8 mm (*n* = 2). General color, in alcohol, dark brown. Head with no modifications. Ocelli 3. Antenna simple, 20-articulated. Maxillary palpus 5-articulated; labial palpus 3-articulated. Mesoscutellum with transverse suture. Metascutellum subtriangular. Anterior femur with small acute apical process. Tibial spur formula 1-3-4. Wing venation reduced in both wings. Abdominal segment VI with pair of internal pouches in posterodorsal area. Ventromesal process on segment VII present. Segment VIII shorter ventrally than dorsally. **Male genitalia**. Segment IX reduced dorsally; sternum subrectangular, with anterior margin rounded (Fig. 15A); in lateral view narrower anteriorly than posteriorly (Fig. 15C). Inferior appendage with peg-like setae, subrectangular in ventral (Fig. 15A) and lateral (Fig. 15C) views; apex with acute corners. Dorsal hook long, almost reaching the inferior appendage apex; in lateral view, with apex slightly broader, almost straight, and truncate (Fig. 15C). Preanal appendage short, rounded and bearing very long setae (Fig. 15B). Subgenital plate apparently absent. Tergum X membranous and rounded (Fig. 15B). Phallus tubular, elongate and slender, slightly constricted mesally; with two curved subapical spines, one short and another one very long; apex rounded and sclerotized; ejaculatory duct sclerotized, sinuous, and not protruding apically (Fig. 15D).

**Holotype.** **BRAZIL: Mato Grosso do Sul:** Bonito, Rio Formosinho, $21\textdegree 1{0}^{^{\prime}}1{6}^{^{\prime}^{\prime}}\mathrm{S}$ $56\textdegree 2{6}^{^{\prime}}4{7}^{^{\prime}^{\prime}}\mathrm{W}$ el. 275 m, 08–13.ix.2013, APM Santos & DM Takiya cols., Malaise trap, male (DZRJ).

**Paratypes.** Same data as holotype, 3 males (DZRJ).

**Etymology.** This species is named in allusion to the river where type specimens were collected.

**Remarks**. This new species appears to be a member of the *campana* group because of internal pouches between abdominal segments VI–VII and phallus with two subapical spines. General aspect of the male genitalia of *Metrichia formosinha* **sp. nov.** is similar to *M*. *forceps* **sp. nov.**, particularly in the inferior appendages with acute corners. However, *M*. *formosinha* **sp. nov.** differs from the latter by dorsal hooks almost straight and capitate (strongly curved and acute in *M*. *forceps* **sp. nov.** and in other species of the *campana* group) and phallus with a very long subapical spine.

COI sequences of *M*. *formosinha* **sp. nov.** showed intraspecific divergences up to 0.8% and minimum interspecific divergences of 24.9% compared to *M*. *talhada* **sp. nov.**

### *Metrichia goiana* sp. nov.

urn:lsid:zoobank.org:act:8726E665-1093-42EF-A1B6-E06178B2DAD1

(Fig. 16)

**Figure 16:**
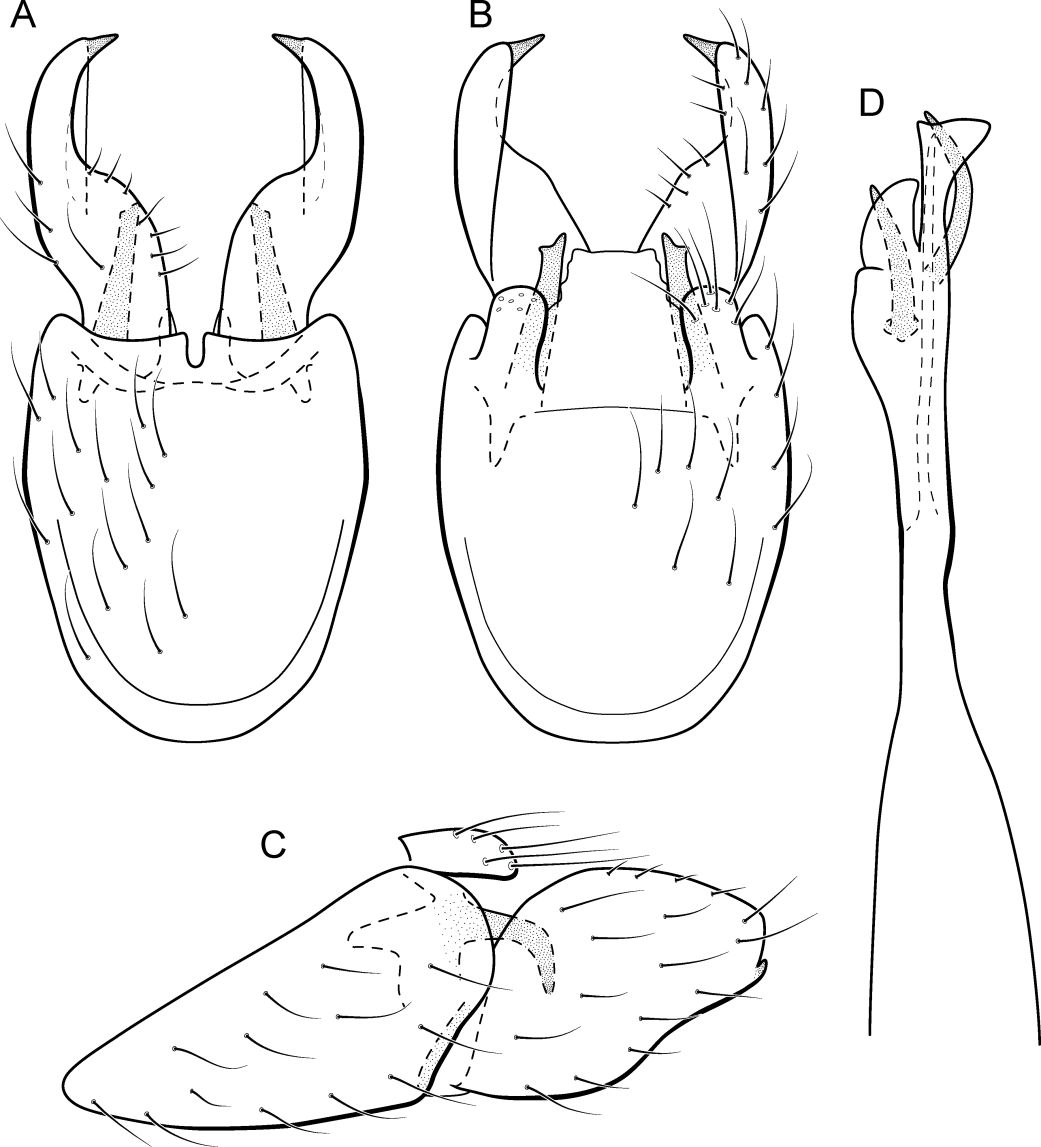
*Metrichia goiana* **sp. nov.**, male genitalia. (A) ventral view; (B) dorsal view; (C) lateral view; (D) phallus, dorsal view.

**Adult male**. Length 1.8–2.0 mm (*n* = 4). General color, in alcohol, dark brown. Head with no modifications. Ocelli 3. Antenna simple, 18-articulated. Maxillary palpus 5-articulated, article IV broad and darkened; labial palpus 3-articulated. Mesoscutellum with transverse suture. Metascutellum subtriangular. Anterior femur with small acute apical process. Tibial spur formula 1-3-4. Wing venation reduced in both wings. Abdominal segment V with pair of internal pouches and pair of dorsolateral brushes; segment VI with a transverse sclerotized plate posteriorly on dorsum; segment VII bearing specialized setae dorsally. Ventromesal process on segment VII absent. Segment VIII shorter ventrally than dorsally. **Male genitalia**. Segment IX reduced dorsally; sternum subrectangular, with anterior margin rounded (Fig. 16A); in lateral view narrower anteriorly than posteriorly (Fig. 16C). Inferior appendage covered by long setae, elongate and narrow in ventral view (Fig. 16A); in lateral view, rounded (Fig. 16A), apex rounded and bearing a tooth-like projection (Fig. 16A). Dorsal hook short, less than half length of inferior appendage; in lateral view, downturned (Fig. 16A). Preanal appendage short and bearing very long setae (Fig. 16B). Subgenital plate apparently absent. Tergum X membranous and truncate (Fig. 16B). Phallus tubular, elongate and slender, slightly constricted mesally; with two long, curved, subapical spines; apex ending into two sclerotized and keel shaped processes; ejaculatory duct sclerotized, straight and not protruding apically (Fig. 16D).

**Holotype.** **BRAZIL: Goiás:** Alto Paraíso de Goiás, Rio Bartolomeu tributary, $14\textdegree 0{7}^{^{\prime}}2{5}^{^{\prime}^{\prime}}\mathrm{S}$ $47\textdegree 3{0}^{^{\prime}}3{0}^{^{\prime}^{\prime}}\mathrm{W}$, el. 1,165 m, 22–25.iii.2013, APM Santos & DM Takiya cols., Malaise trap, male (DZRJ).

**Paratypes.** Same data as holotype, 2 males (MNRJ), 1 male (DZRJ).

**Etymology.** The species is named in reference to Goiás State. “Goiana” is a Portuguese adjective for people from Goiás.

**Remarks**. This is another member of *nigritta* group. Male genitalia of this species are similar to *M*. *potosina* Bueno-Soria, 2002 and *M*. *ubajara* **sp. nov.**, due to rounded and elongate inferior appendages in lateral view. This new species differs from *M*. *ubajara* **sp. nov.** by the presence of an apical tooth on inferior appendages, also present in *M. potosina*. *Metrichia goiana* **sp. nov.** can be distinguished from *M*. *potosina* by the two long subapical spines on phallus, whereas *M*. *potosina* has three.

### *Metrichia itabaiana* sp. nov.

urn:lsid:zoobank.org:act:1C902E75-7ECD-4875-A680-6440A3E5E9E9

(Figs. 17 and 26B)

**Figure 17:**
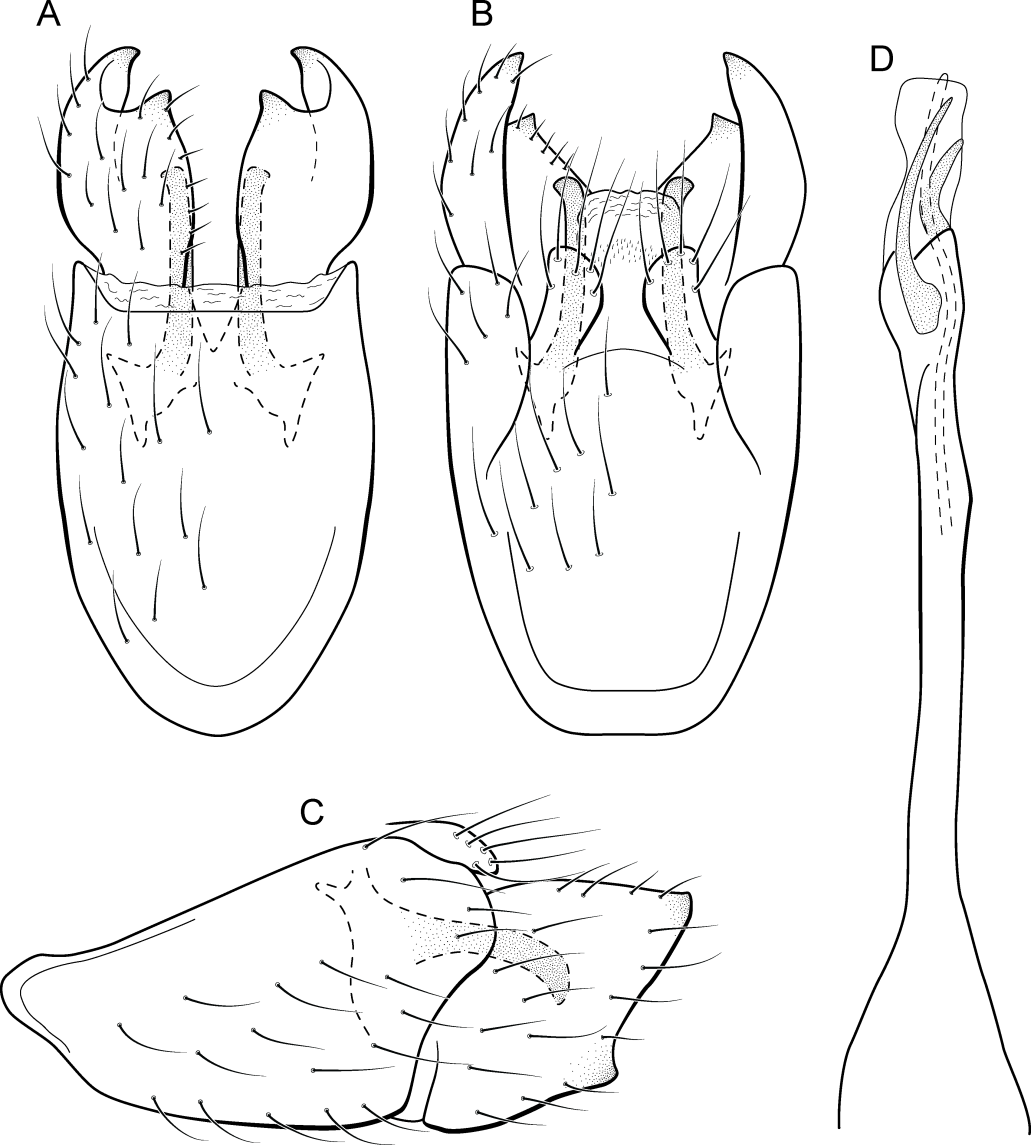
*Metrichia itabaiana* **sp. nov.**, male genitalia. (A) ventral view; (B) dorsal view; (C) lateral view; (D) phallus, dorsal view.

**Adult male**. Length 1.8–2.1 mm (*n* = 3). General color, in alcohol, brown. Head with no modifications. Ocelli 3. Antenna simple, 20-articulated. Maxillary palpus 5-articulated; labial palpus 3-articulated. Mesoscutellum with transverse suture. Metascutellum subtriangular. Anterior femur without processes. Tibial spur formula 1-3-4. Wing venation reduced in both wings. Abdominal segment V ventrally with a mesal brush of long setae; segment VI with tergum as a sclerotized triangular plate surrounded by specialized setae (Fig. 26B), internally with pair of internal pouches; segment VII bearing specialized setae ventrally and dorsally (Fig. 26B). Ventromesal process on segment VII present. Segment VIII shorter ventrally than dorsally and bearing a brush of long setae dorsally. **Male genitalia**. Segment IX reduced dorsally; sternum subpentagonal (Fig. 17A); in lateral view narrower anteriorly than posteriorly (Fig. 17C). Inferior appendage short, covered by long setae (Fig. 17A); in lateral view, subtrapezoidal (Fig. 17C), apex excavated and with two acute and sclerotized process (Figs. 17A and 17B). Dorsal hook short, almost half length of inferior appendage; in lateral view, slightly downturned (Fig. 17C). Preanal appendage elongate, but shorter than inferior appendage, and bearing very long setae (Fig. 17B). Subgenital plate apparently absent. Tergum X membranous and truncate (Fig. 17B). Phallus tubular, elongate and slender, slightly constricted mesally; with two long, curved, subapical spines; apex truncate and slightly sclerotized; ejaculatory duct sclerotized, sinuous, and protruding apically (Fig. 17D).

**Holotype. Sergipe:** Areia Branca, Parque Nacional da Serra de Itabaiana, Rio dos Negros, $10\textdegree 4{4}^{^{\prime}}5{1}^{^{\prime}^{\prime}}\mathrm{S}$ $37\textdegree 2{0}^{^{\prime}}2{4}^{^{\prime}^{\prime}}\mathrm{W}$, el. 208 m, 17.vi.2014, APM Santos, DM Takiya & WRM Souza cols., light trap, male (DZRJ).

**Paratypes.** Same data as holotype, 1 male (DZRJ), 1 male (MZUFBA); same data, except Riacho Água Fria, $10\textdegree 4{5}^{^{\prime}}1{7}^{^{\prime}^{\prime}}\mathrm{S}$ $37\textdegree 2{0}^{^{\prime}}3{2}^{^{\prime}^{\prime}}\mathrm{W}$, el. 196 m, 17–19.vi.2014, APM Santos, DM Takiya, WRM Souza cols., Malaise trap, 2 males (MNRJ). **Goiás:** Alto Paraíso, Rio Bartolomeu tributary, $14\textdegree 0{7}^{^{\prime}}2{5}^{^{\prime}^{\prime}}\mathrm{S}$ $47\textdegree 3{0}^{^{\prime}}3{0}^{^{\prime}^{\prime}}\mathrm{W}$, el. 1,165 m, 22–25.iii.2013, APM Santos & DM Takiya cols., Malaise trap, 1 male (DZRJ).

**Etymology.** This species is named in reference to Serra de Itabaiana, Sergipe, where the holotype was collected.

**Remarks.** This new species appears to be a member of the *campana* group because of internal pouches between abdominal segments VI and VII and pair of long subapical spines on phallus, but it lacks the acute process on the mesal area of phallus. Male genitalia of *M*. *itabaiana* **sp. nov.** resemble those of *M*. *campana* and *M. vulgaris* **sp. nov.**, particularly, in the excavated inferior appendages, with acute and darkened corners. However, the new species can be recognized by very long curved subapical spines on phallus and subtrapezoidal aspect of inferior appendages in lateral view.

Only two COI sequences were obtained for *M*. *itabaiana* **sp. nov.**, one from Sergipe (Northeastern Brazil) and another from Goiás (Centralwestern Brazil). The COI divergence between these two samples was 1.9% and minimum interspecific distance was 19.4% in relation to *M*. *rafaeli* **sp. nov.**, which belongs to a different species group based on morphological features.

### *Metrichia longissima* sp. nov.

urn:lsid:zoobank.org:act:F87C549F-6F84-4466-AFCE-940729F32F46

(Figs. 18 and 26C)

**Figure 18:**
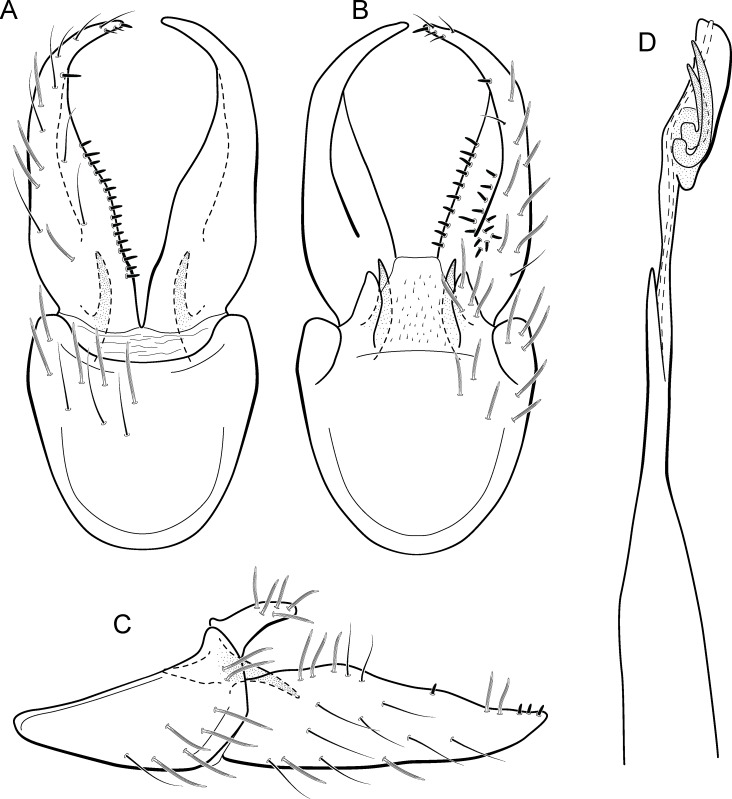
*Metrichia longissima* **sp. nov.**, male genitalia. (A) ventral view; (B) dorsal view; (C) lateral view; (D) phallus, dorsal view.

**Adult male**. Length 2.5–2.7 mm (*n* = 2). General color, in alcohol, brown. Head with no modifications. Ocelli 3. Antenna simple, 18-articulated. Maxillary palpus 5-articulated; labial palpus 3-articulated. Mesoscutellum with transverse suture. Metascutellum subtriangular. Anterior femur with small acute apical process. Tibial spur formula 1-3-4. Wing venation reduced in both wings. Abdominal segment IV with dorsal area expanded posteriorly bearing stout setae; segment VI with stout and striate setae (Fig. 26C); segment VII with stout and striate setae (Fig. 26C). Ventromesal process on segment VII absent. Segment VIII shorter ventrally than dorsally. **Male genitalia**. Segment IX reduced dorsally; sternum subpentagonal (Fig. 18A); in lateral view narrower anteriorly than posteriorly (Fig. 18C). Inferior appendage bearing scale-like setae, very elongate; in ventral view, curved inward apically (Fig. 18A); in lateral view, tapering to a rounded apex (Fig. 18C). Dorsal hook short and straight; in lateral view, slightly downturned (Fig. 18C). Preanal appendage elongate, but shorter than half length of inferior appendage, and bearing stout and striate setae (Fig. 18B). Subgenital plate apparently absent. Tergum X membranous and truncate (Fig. 18B). Phallus tubular, elongate and slender, slightly constricted mesally, with a median process; with two long, curved, subapical spines; apex rounded and sclerotized; ejaculatory duct sclerotized and protruding apically (Fig. 18D).

**Holotype.** **BRAZIL: Rio de Janeiro:** Itatiaia, Rio Palmital, $22\textdegree 2{5}^{^{\prime}}3{4}^{^{\prime}^{\prime}}\mathrm{S}$ $44\textdegree 3{2}^{^{\prime}}5{2}^{^{\prime}^{\prime}}\mathrm{W}$, el. 637 m, 07.iii.2008, LL Dumas, JL Nessimian & MR de Souza cols., light trap, male (DZRJ).

**Paratype.** **Brazil: Rio de Janeiro:** Teresópolis, Parque Nacional da Serra dos Órgãos, Rio Paquequer, $22\textdegree 2{7}^{^{\prime}}2{5}^{^{\prime}^{\prime}}\mathrm{S}$ $42\textdegree 5{9}^{^{\prime}}5{2}^{^{\prime}^{\prime}}\mathrm{W}$, el. 1,100 m, 15–18.ix.2011, APM Santos, DM Takiya, BM Vasconcelos & RA Carvalho cols., Malaise trap, 1 male (MNRJ).

**Etymology.** The species name is an allusion to the elongate inferior appendages, unusual for *Metrichia* species.

**Remarks**. The new species is most similar to *M*. *sesquipedalis* Bueno-Soria & Holzenthal, 2003, sharing with it very long inferior appendages with very short dorsal hooks. The new species is easily distinguished from the latter by their internal pouches in the male abdominal segment VI and phallus with only two subapical spines (three in *M. sesquipedalis*).

Two COI sequences were generated for *M*. *longissima* **sp. nov.**, one from a specimen from Itatiaia and the other from Teresópolis in Rio de Janeiro State, localities in distinct mountain ranges, Serra da Mantiqueira and Serra do Mar, respectively. The genetic distance between sequences of *M*. *longissima* **sp. nov.** was 0.4%. The minimum interspecific distance was found to *M*. *itabaiana* **sp. nov.** with 21.5% divergence.

### *Metrichia peluda* sp. nov.

urn:lsid:zoobank.org:act:E1B7E1AE-5751-4D10-9B07-47A8CF849C7F

(Figs. 19 and 26D)

**Figure 19:**
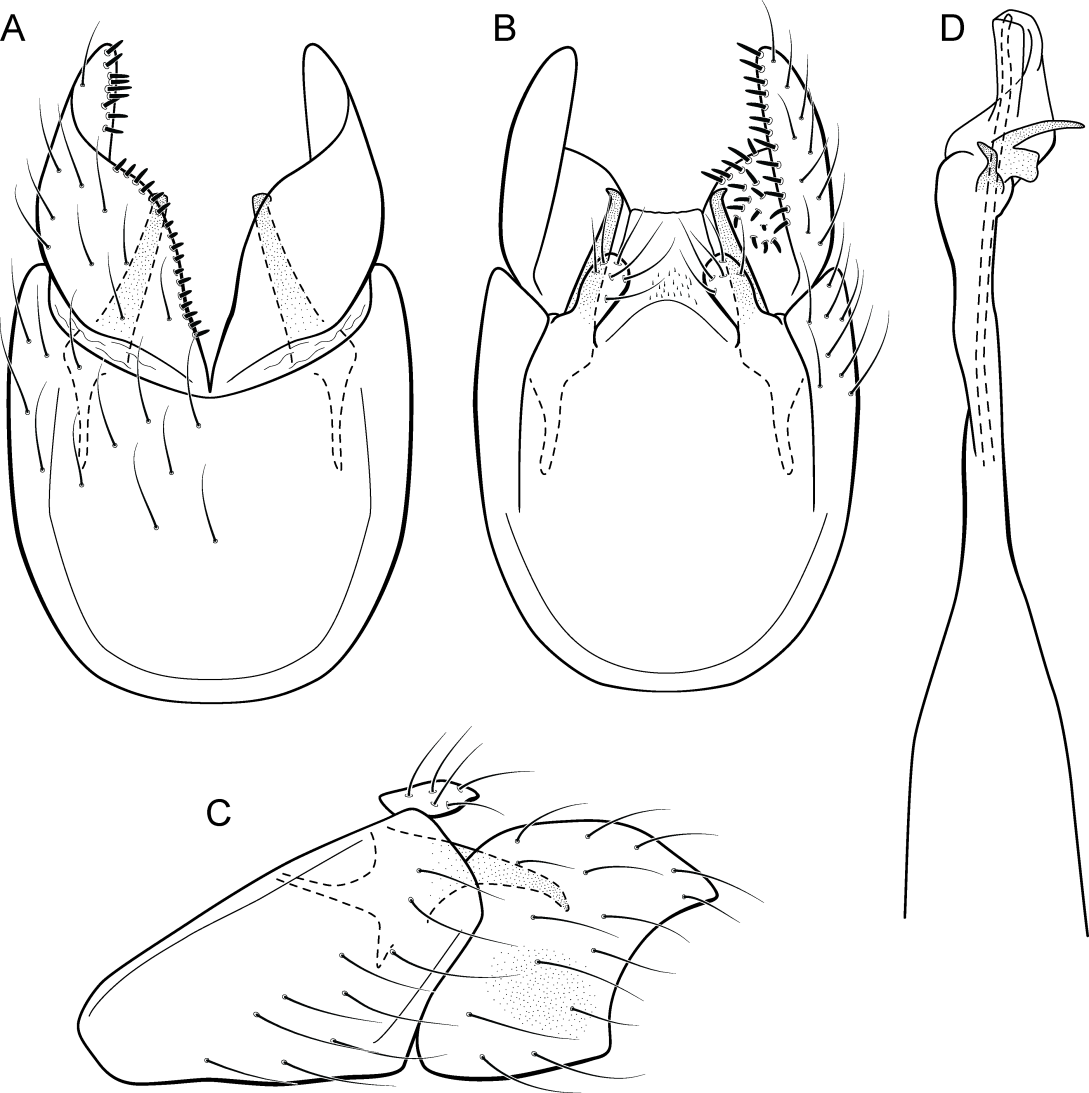
*Metrichia peluda* **sp. nov.**, male genitalia. (A) ventral view; (B) dorsal view; (C) lateral view; (D) phallus, dorsal view.

**Adult male**. Length 2.7–3.0 mm (*n* = 3). General color, in alcohol, dark brown. Head with no modifications. Ocelli 3. Antenna simple, 18-articulated. Maxillary palpus 5-articulated; labial palpus 3-articulated. Mesoscutellum with transverse suture. Metascutellum subtriangular. Anterior femur without processes. Tibial spur formula 1-3-4. Wing venation reduced in both wings. Abdominal segment V with dorsolateral brushes of long setae; segment VI with dorsolateral brushes of long setae (Fig. 26D). Ventromesal process on segment VII present. Segment VIII shorter ventrally than dorsally. **Male genitalia**. Segment IX reduced dorsally; sternum subrectangular, with anterior margin rounded (Fig. 19A); in lateral view narrower anteriorly than posteriorly (Fig. 19C). Inferior appendage covered by long setae, subtrapezoidal in ventral view (Fig. 19A); apex excavated; in lateral view, rounded (Fig. 19C). Dorsal hook short, almost half length of inferior appendage; in lateral view, slightly downturned (Fig. 19C). Preanal appendage short and bearing very long setae (Fig. 19B). Subgenital plate apparently absent. Tergum X membranous and truncate (Fig. 19B). Phallus tubular, elongate and slender, slightly constricted mesally; with two curved subapical spines, one short and another long; apex rounded and folded; ejaculatory duct sclerotized, straight and protruding apically (Fig. 19D).

**Holotype**. **BRAZIL: Rio de Janeiro:** Itatiaia, 1st order tributary of Rio Palmital, $22\textdegree 2{5}^{^{\prime}}4{0}^{^{\prime}^{\prime}}\mathrm{S}$ $44\textdegree 3{2}^{^{\prime}}4{6}^{^{\prime}^{\prime}}\mathrm{W}$, el. 584 m, 07.iii.2008, JL Nessimian, LL Dumas & MR de Souza cols., light trap, male (DZRJ).

**Paratypes.** Same data as holotype, 1 male (MNRJ); same data, except Rio Palmital, $22\textdegree 2{5}^{^{\prime}}3{4}^{^{\prime}^{\prime}}\mathrm{S}$ $44\textdegree 3{2}^{^{\prime}}5{2}^{^{\prime}^{\prime}}\mathrm{W}$, el. 637 m, 07.iii.2008, LL Dumas, JL Nessimian & MR de Souza cols., light trap, 4 males (DZRJ).

**Etymology.** The name of this species refers to dense brushes of setae on the dorsal area of the male abdomen. In Portuguese “peluda” means “hairy.”

**Remarks.** Modifications on male abdominal segments V, VI, and VII suggest that this new species belongs to the *campana* group. The general aspect of inferior appendages is somewhat similar to *M*. *forceps* **sp. nov.** and *M*. *formosinha* **sp. nov.**, which are excavate posteriorly. However, *M*. *peluda* **sp. nov.** is readily identified by the dense brushes of setae on the dorsum of abdominal segments V, VI, and VII. Besides, the male genitalia of this new species differ from those described for *M*. *forceps* **sp. nov.** and *M*. *formosinha* **sp. nov.** by the rounded corners of inferior appendages instead of acute and by phallus with two subapical spines with unequal sizes.

### *Metrichia rafaeli* sp. nov.

urn:lsid:zoobank.org:act:CBCADBB8-2C79-49AB-8345-CC1E6FA2AEE9

(Fig. 20)

**Figure 20:**
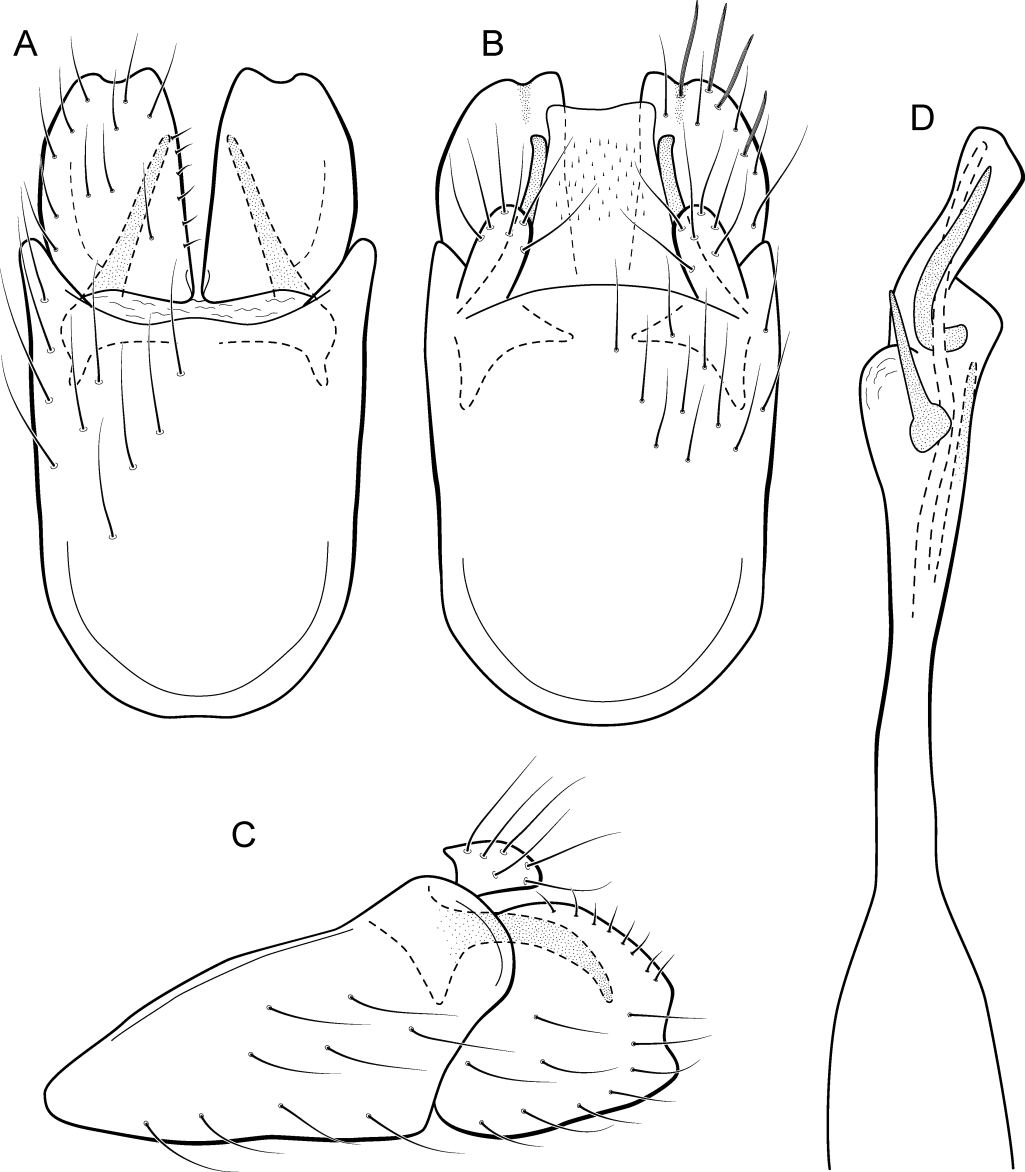
*Metrichia rafaeli* **sp. nov.**, male genitalia. (A) ventral view; (B) dorsal view; (C) lateral view; (D) phallus, dorsal view.

**Adult male**. Length 2.0–2.5 mm (*n* = 4). General color, in alcohol, dark brown. Head with no modifications. Ocelli 3. Antenna simple, 20-articulated. Maxillary palpus 5-articulated; labial palpus 3-articulated. Mesoscutellum with transverse suture. Metascutellum subtriangular. Anterior femur without processes. Tibial spur formula 1-3-4. Wing venation reduced in both wings. Abdominal segment V with pair of internal pouches; segment VI with pair of internal pouches and pair of lateral external sacs with specialized setae; segment VII bearing specialized setae dorsally. Ventromesal process on segment VII present. Segment VIII shorter ventrally than dorsally. **Male genitalia**. Segment IX reduced dorsally; sternum subrectangular, with anterior margin rounded (Fig. 20A); in lateral view narrower anteriorly than posteriorly (Fig. 20C). Inferior appendage covered by long setae, short and rounded, with apex slightly excavated (Fig. 20A); in lateral view, rounded (Fig. 20C). Dorsal hook long, more than half length of inferior appendage; in lateral view, downturned (Fig. 20C). Preanal appendage elongate, but shorter than half length of inferior appendage, and bearing stout and striate setae (Fig. 20B). Subgenital plate apparently absent. Tergum X membranous and truncate (Fig. 20B). Phallus tubular, elongate and slender, slightly constricted mesally, with a median process; with two long, curved, subapical spines, and a membranous lobe; apex rounded and sclerotized; ejaculatory duct sclerotized, sinuous, and not protruding apically (Fig. 20D).

**Holotype male.** **BRAZIL: Ceará:** Ubajara, Parque Nacional de Ubajara, Rio das Minas, $03\textdegree 5{0}^{^{\prime}}0{3}^{^{\prime}^{\prime}}\mathrm{S}$ $40\textdegree 5{4}^{^{\prime}}1{8}^{^{\prime}^{\prime}}\mathrm{W}$, el. 524, 17–18.ii.2013, DM Takiya, JA Rafael, RR Cavichioli & APM Santos cols., Malaise trap (CZMA).

**Paratypes.** Same data as holotype, 1 male (MZUFBA); same data, except Rio das Minas, $03\textdegree 4{9}^{^{\prime}}5{8}^{^{\prime}^{\prime}}\mathrm{S}$ $40\textdegree 5{3}^{^{\prime}}5{3}^{^{\prime}^{\prime}}\mathrm{W}$, el. 420 m, 20–23.iv.2012, F Limeira-de-Oliveira et al.cols., Malaise trap, 1 male (CZMA); same data, except 14–16.ii.2013, DM Takiya, JA Rafael, RR Cavichioli & APM Santos cols., 1 male (DZRJ).

**Etymology.** This species is named in honor of the Brazilian entomologist Dr. José Albertino Rafael (INPA), who has collected many interesting caddisflies, including some species described here.

**Remarks**. This new species belongs to the *nigritta* group due to internal pouches between abdominal segments V and VI and long and acute process on phallus. The male genitalia of *M*. *rafaeli* **sp. nov.** are more similar to *M*. *magna* Bueno-Soria & Holzenthal, 2003 with short and simple inferior appendages. However, the new species can be easily distinguished from the latter and other *Metrichia* species in this group by the posterior margin of inferior appendages slightly excavated and very long subapical spines of phallus.

Although specimens with barcode sequences of *M*. *rafaeli* **sp. nov.** were collected at the same locality, haplotypes were not identical having intraspecific divergence of 0.4%. The lowest interspecific distance was 19.4% when compared with *M*. *itabaiana* **sp. nov.**

### *Metrichia simples* sp. nov.

urn:lsid:zoobank.org:act:05933E6E-A6FF-49DE-982E-54B263830D10

(Figs. 21 and 26E)

**Figure 21:**
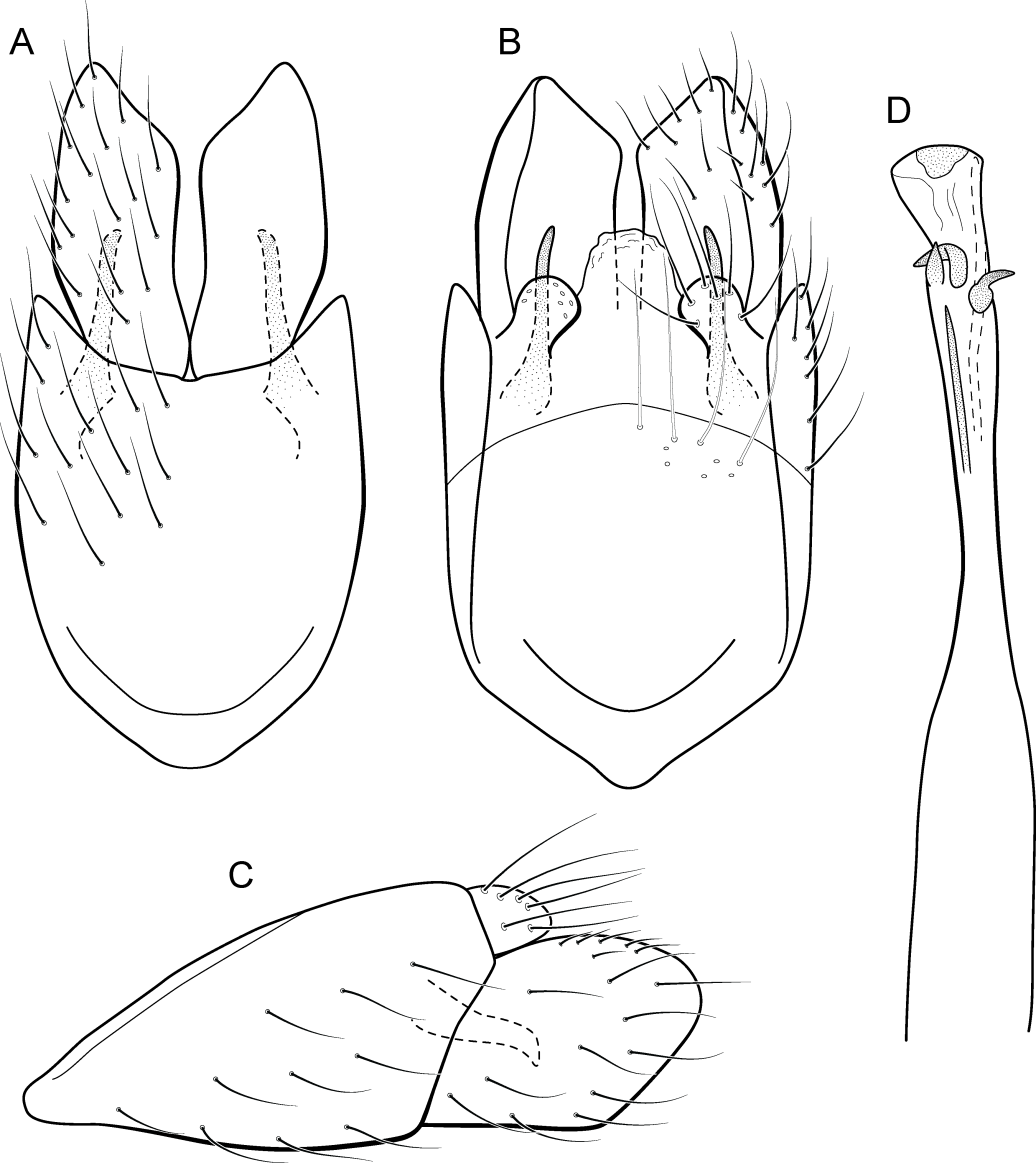
*Metrichia simples* **sp. nov.**, male genitalia. (A) ventral view; (B) dorsal view; (C) lateral view; (D) phallus, dorsal view.

**Adult male**. Length 2.1–2.2 mm (*n* = 2). General color, in alcohol, brown. Head with no modifications. Ocelli 3. Antenna simple, 19-articulated. Maxillary palpus 5-articulated; labial palpus 3-articulated. Mesoscutellum with transverse suture. Metascutellum subtriangular. Anterior femur without processes. Tibial spur formula 1-3-4. Wing venation reduced in both wings. Abdominal segment IV with dorsal area expanded posteriorly bearing stout setae; segment VI bearing very long setae laterally, with a brush of short setae covered dorsally by a triangular plate (Fig. 26E); segment VII with a brush of short setae dorsally (Fig. 26E). Ventromesal process on segment VII present. Segment VIII shorter ventrally than dorsally. **Male genitalia**. Segment IX reduced dorsally; sternum subpentagonal (Fig. 21A); in lateral view narrower anteriorly than posteriorly (Fig. 21C). Inferior appendage covered by long setae, with apex obliquely truncate; subtrapezoidal in ventral view (Fig. 21A); in lateral view, subtrapezoidal (Fig. 21C). Dorsal hook short, almost half length of inferior appendage; in lateral view, slightly downturned (Fig. 21C). Preanal appendage short, rounded and bearing very long setae (Fig. 21B). Subgenital plate apparently absent. Tergum X membranous and rounded (Fig. 21B). Phallus tubular, elongate and slender, slightly constricted mesally; with four subapical spines, three short and one long and straight; apex rounded with a small sclerite; ejaculatory duct sclerotized and not protruding apically (Fig. 21D).

**Holotype.** **BRAZIL: Paraná:** Céu Azul, Parque Nacional do Iguaçu, Rio Azul, $25\textdegree 0{9}^{^{\prime}}2{1}^{^{\prime}^{\prime}}\mathrm{S}$ $53\textdegree 4{7}^{^{\prime}}4{4}^{^{\prime}^{\prime}}\mathrm{W}$, el. 510 m, 6–8 ix.2012, APM Santos, DM Takiya, ALH Oliveira, GA Jardim & BHL Sampaio cols., Malaise trap, male (DZRJ).

**Paratypes.** Same data as holotype, 1 male (MNRJ).

**Etymology.** This species is named in reference to the simple aspect of the male genitalia and abdomen, without modifications and processes seen in other *Metrichia* species.

**Remarks**. This species can be assigned to the *campana* group because of pouches in abdominal segments VI and VII. The new species shares the general aspect of the genitalia with *M*. *quadrata*, particularly, in the quadrangular or rectangular shape of inferior appendages and the short subapical spines on phallus. *Metrichia simples* **sp. nov.** can be recognized by the short dorsal hook, reaching up to midlength of inferior appendages (subequal to inferior appendages in *M*. *quadrata*), obliquely truncate apex of the inferior appendages, and strongly curved spines on phallus.

### *Metrichia talhada* sp. nov.

urn:lsid:zoobank.org:act: 5456FEB8-5193-46DD-A10F-9FFADFCB59EC

(Fig. 22)

**Figure 22:**
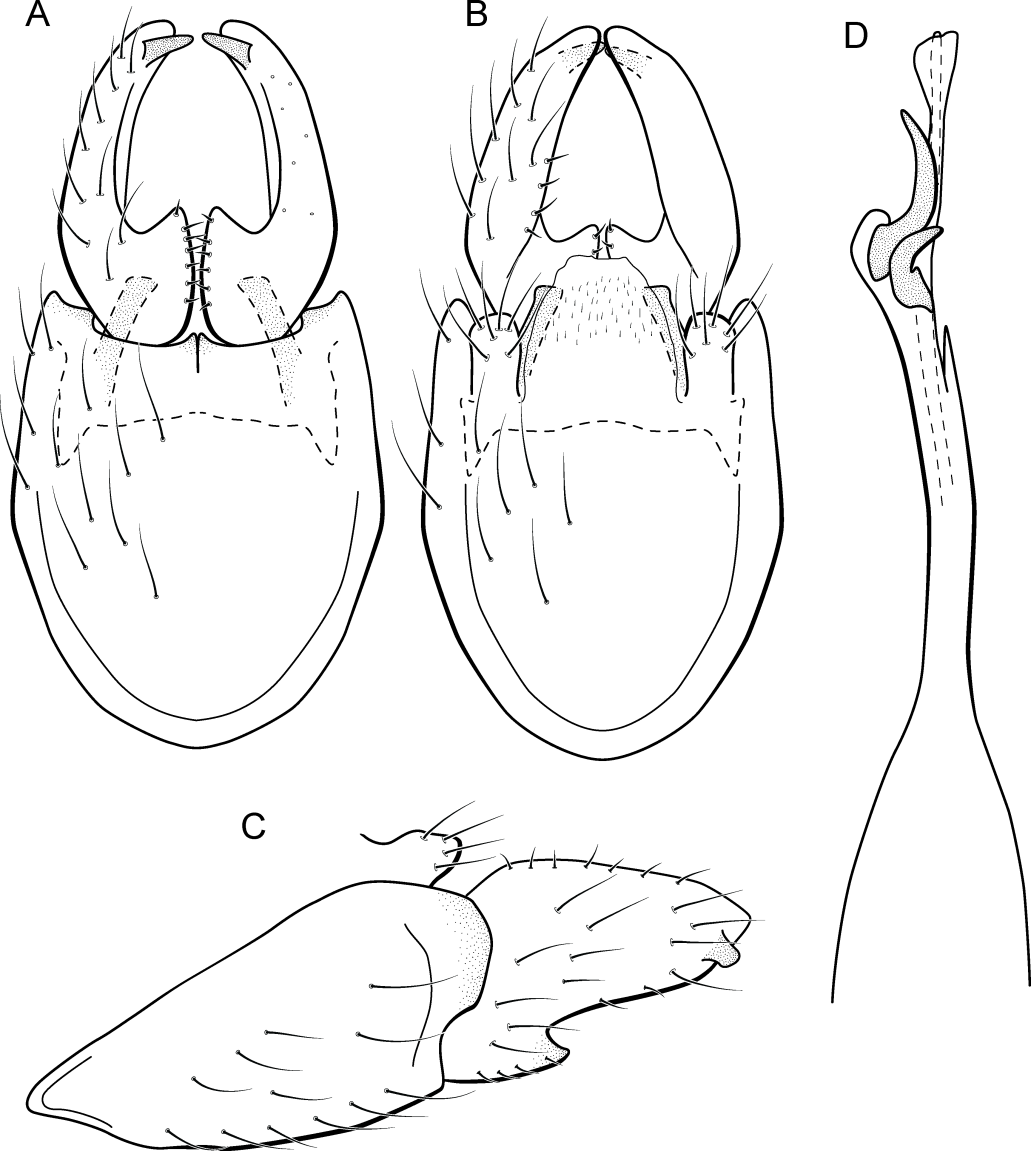
*Metrichia talhada* **sp. nov.**, male genitalia. (A) ventral view; (B) dorsal view; (C) lateral view; (D) phallus, dorsal view.

**Adult male**. Length 1.8–2.0 mm (*n* = 10). General color, in alcohol, brown. Head with no modifications. Ocelli 3. Antenna simple, 18-articulated. Maxillary palpus 5-articulated; labial palpus 3-articulated. Mesoscutellum with transverse suture. Metascutellum subtriangular. Anterior femur with small acute apical process. Tibial spur formula 1-3-4. Wing venation reduced in both wings. Abdominal segment V with pair of internal pouches and pair of dorsolateral brushes; segment VI with dorsolateral brushes of long setae; segment VII bearing specialized setae dorsally. Ventromesal process on segment VII absent. Segment VIII shorter ventrally than dorsally. **Male genitalia**. Segment IX reduced dorsally; sternum subpentagonal (Fig. 22A); in lateral view, narrower anteriorly than posteriorly (Fig. 22C). Inferior appendage elongate, apex rounded and bearing a tooth-like projection; with a deep C-shaped notch in ventral view (Fig. 22A); in lateral view, with an acute projection (Fig. 22C). Dorsal hook short, less than half length of inferior appendage (Fig. 22A); in lateral view, downturned. Preanal appendage short, rounded and bearing very long setae (Fig. 22B). Subgenital plate apparently absent. Tergum X membranous and truncate (Fig. 22B). Phallus tubular, elongate and slender, slightly constricted mesally, with a median process; with two curved subapical spines, one short and another long; apex rounded and sclerotized; ejaculatory duct sclerotized, straight and protruding apically (Fig. 22D).

**Holotype.** **BRAZIL: Alagoas:** Quebrangulo, Reserva Biológica de Pedra Talhada, Rio Caranguejo, $09\textdegree 1{5}^{^{\prime}}2{6}^{^{\prime}^{\prime}}\mathrm{S}$ $36\textdegree 2{5}^{^{\prime}}0{8}^{^{\prime}^{\prime}}\mathrm{W}$, el. 550 m, 19–28.vi.2014, APM Santos, DM Takiya, WRM Souza cols., Malaise trap, male (DZRJ).

**Paratypes.** Same data as holotype, 8 males (DZRJ), 7 males (MZUFBA).

**Etymology.** This species is named in reference to its type locality, the Reserva Biológica de Pedra Talhada.

**Remarks**. The new species belongs to the *nigritta* group because of their internal pouches between abdominal segments V and VI and phallus with two subapical curved spines and an acute process near mesal area. *Metrichia talhada* **sp. nov.** shares the apical tooth on inferior appendages with *M*. *potosina*, *M*. *goiana* **sp. nov.**, and *Metrichia tere* **sp. nov.** From *M*. *potosina* and *M*. *goiana* **sp. nov.**, it is easily distinguished by the shape of the inferior appendages, with a deep C-shaped notch on ventral margin and subapical tooth stout and slightly truncate in lateral view, and subapical spines on phallus, one long and another short. Although the male genitalia of *M. talhada* **sp. nov.** are very similar to *M. tere* **sp. nov.**, these two species can be separated by the shape of inferior appendages teeth, each slightly truncate and subapical in the former and acute and apical in the later species.

Sequences of *M*. *talhada* **sp. nov.** showed 0.0% of divergence. Morphological similarity between this species and its sister (*M*. *tere* **sp. nov.**) had a relatively low genetic divergence of 12.6%, the lowest interspecific distance found in our sampling. In addition to minor but stable differences in genital structures, all molecular analyses with COI sequences (NJ, ABGD, GMYC) corroborate the distinction between *M*. *talhada* **sp. nov.** and *M*. *tere* **sp. nov.**, which are formally described here as different species.

### *Metrichia tere* sp. nov.

urn:lsid:zoobank.org:act:21376F9C-6308-47BA-ADB8-A42848AD8FB5

(Fig. 23)

**Figure 23:**
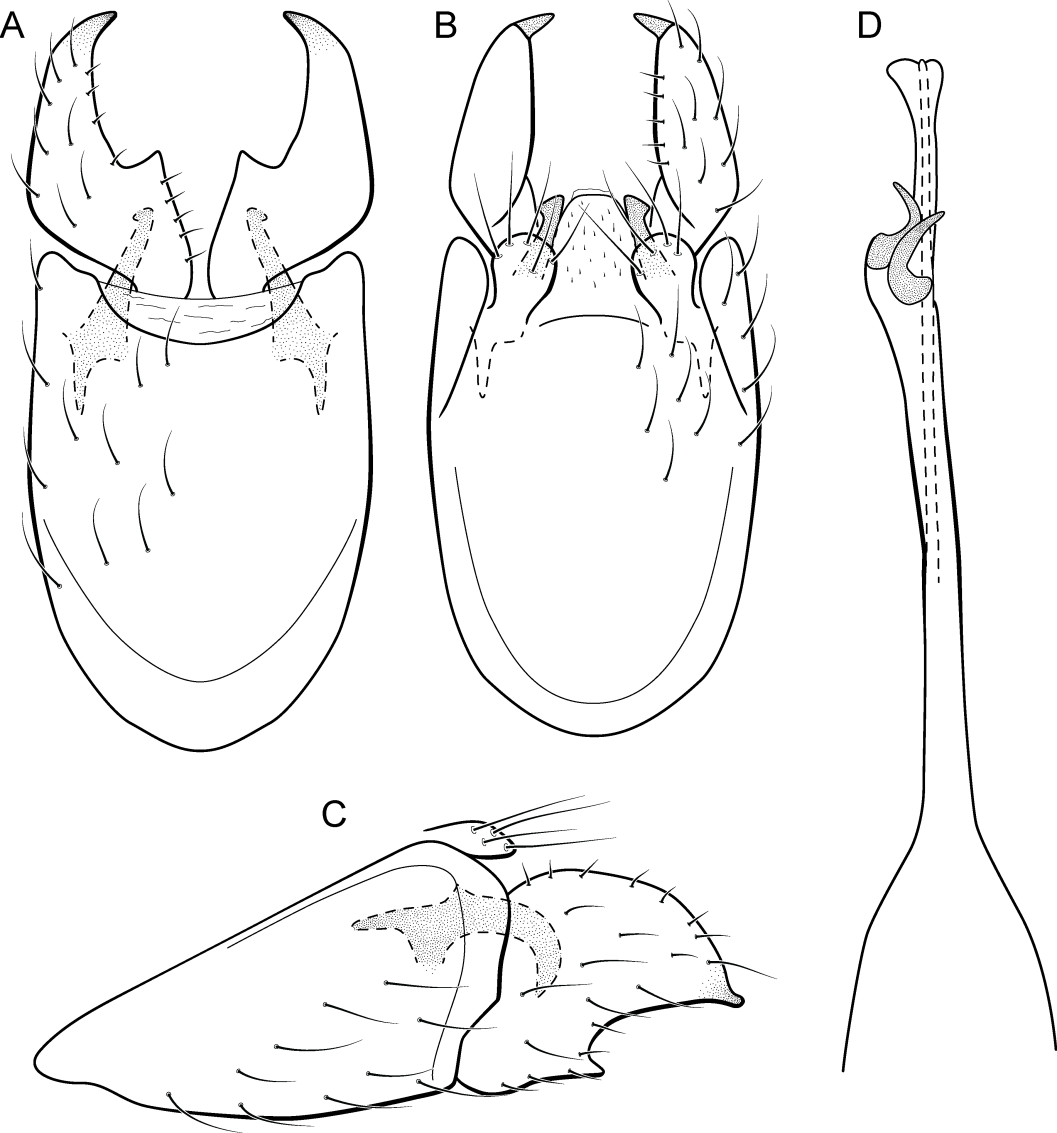
*Metrichia tere* **sp. nov.**, male genitalia. (A) ventral view; (B) dorsal view; (C) lateral view; (D) phallus, dorsal view.

**Adult male**. Length 1.9–2.1 mm (*n* = 10). General color, in alcohol, brown. Head with no modifications. Ocelli 3. Antenna simple, 18-articulated. Maxillary palpus 5-articulated; labial palpus 3-articulated. Mesoscutellum with transverse suture. Metascutellum subtriangular. Anterior femur with small acute apical process. Tibial spur formula 1-3-4. Wing venation reduced in both wings. Abdominal segment V with pair of internal pouches and pair of dorsolateral brushes; segment VI with dorsolateral brushes of long setae; segment VII bearing specialized setae dorsally. Ventromesal process on segment VII absent. Segment VIII shorter ventrally than dorsally. **Male genitalia**. Segment IX reduced dorsally; sternum subpentagonal (Fig. 23A); in lateral view, narrower anteriorly than posteriorly (Fig. 23C). Inferior appendage elongate, apex with an acute projection; with a deep C-shaped notch in ventral view (Fig. 23A); in lateral view, with an acute projection (Fig. 23C). Dorsal hook short, less than half length of inferior appendage; in lateral view, downturned (Fig. 23C). Preanal appendage short, rounded and bearing very long setae (Fig. 23B). Subgenital plate apparently absent. Tergum X membranous and truncate (Fig. 23B). Phallus tubular, elongate and slender, slightly constricted mesally, with a median process; with two curved subapical spines, one short and another long; apex rounded and sclerotized; ejaculatory duct sclerotized, straight and protruding apically (Fig. 23D).

**Holotype.** **BRAZIL: Rio de Janeiro:** Teresópolis, Parque Nacional da Serra dos Órgãos, Rio Paquequer, $22\textdegree 2{7}^{^{\prime}}2{5}^{^{\prime}^{\prime}}\mathrm{S}$ $42\textdegree 5{9}^{^{\prime}}5{2}^{^{\prime}^{\prime}}\mathrm{W}$, el. 1,100 m, 15–18.ix.2011, APM Santos, DM Takiya, BM Vasconcelos & RA Carvalho cols., Malaise trap, male (DZRJ).

**Paratypes.** Same data as holotype, 9 males (MNRJ), 19 males (DZRJ).

**Etymology.** This species is named in reference to the city of Teresópolis (= “City of Teresa”), affectionately called as “Terê”. The city was named in honor of Teresa Cristina, Brazilian Empress from 1843 to 1889, wife of Dom Pedro II.

**Remarks.** This new species is very similar to the preceding one, also belonging to the *nigritta* group. *Metrichia tere* **sp. nov.** can be distinguished from *M*. *talhada* **sp. nov.** by inferior appendages with an apical acute projection, whereas in *M*. *talhada* **sp. nov.** inferior appendages have a subapical tooth, which is more truncate than acute.

Molecular data agree with the morphological distinction of *M*. *tere* **sp. nov.**, as commented on above. Intraspecific K2P divergence among specimens sampled (*n* = 3) of this species was 0.0%, and as mentioned in the previous description, *M*. *talhada* **sp. nov.** and *M*. *tere* **sp. nov.** showed the lowest observed interspecific distance (12.6%).

### *Metrichia ubajara* sp. nov.

urn:lsid:zoobank.org:act:68FD2A24-BC85-42A3-9007-96A42657DAE3

(Fig. 24)

**Figure 24:**
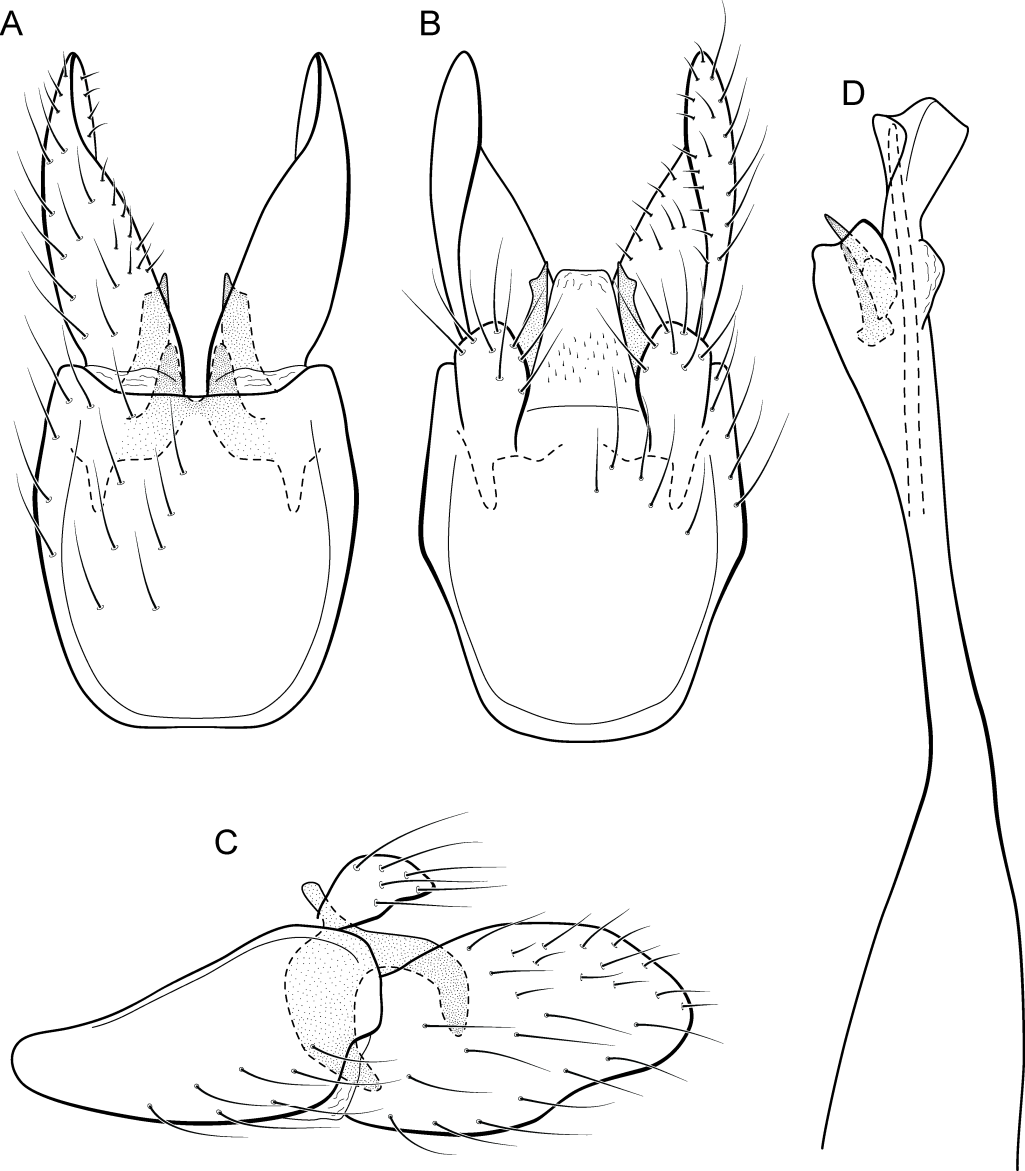
*Metrichia ubajara* **sp. nov.**, male genitalia. (A) ventral view; (B) dorsal view; (C) lateral view; (D) phallus, dorsal view.

**Adult male**. Length 2.0–2.7 mm (*n* = 11). General color, in alcohol, brown. Head with no modifications. Ocelli 3. Antenna simple, 18-articulated. Maxillary palpus 5-articulated; labial palpus 3-articulated. Mesoscutellum with transverse suture. Metascutellum subtriangular. Anterior femur without processes. Tibial spur formula 1-3-4. Wing venation reduced in both wings. Abdominal segment V with pair of internal pouches and median internal plate in posterior region; with specialized setae on dorsum; segment VI with pair of internal pouches in posterodorsal area. Ventromesal process on segment VII absent. Segment VIII shorter ventrally than dorsally. **Male genitalia**. Segment IX reduced dorsally; sternum subrectangular (Fig. 24A); in lateral view, narrower anteriorly than posteriorly (Fig. 24C). Inferior appendage covered by long setae, apex rounded; elongate and narrow in ventral view (Fig. 24A); in lateral view, rounded (Fig. 24C). Dorsal hook short, less than half length of inferior appendage; apex downturned; basally with a wide and sclerotized projection; in lateral view, C-shaped (Fig. 24C). Preanal appendage elongate, but shorter than half length of inferior appendage, and bearing stout and striate setae (Fig. 24B). Subgenital plate apparently absent. Tergum X membranous and truncate (Fig. 24B). Phallus tubular, elongate and slender, slightly constricted mesally; with two long, curved, subapical spines; apex rounded and folded; ejaculatory duct sclerotized, sinuous, and protruding apically (Fig. 24D).

**Holotype.** **BRAZIL: Ceará:** Ubajara, Parque Nacional de Ubajara, Rio das Minas, $03\textdegree 4{9}^{^{\prime}}5{8}^{^{\prime}^{\prime}}\mathrm{S}$ $40\textdegree 5{3}^{^{\prime}}5{3}^{^{\prime}^{\prime}}\mathrm{W}$, el. 420 m, 20–23.iv.2012, DM Takiya, JA Rafael, F Limeira-de-Oliveira et al. cols., Malaise trap, male (CZMA).

**Paratypes.** Same data as holotype, 1 male (CZMA); same data, except 13–17.ix.2012, 25 males (CZMA), 12 males (DZRJ); same data, except 18–30.xi.2012, 9 males (INPA); same data, except 14–16.ii.2013, 5 males (MZUFBA); same data, except Rio das Minas, $03\textdegree 5{0}^{^{\prime}}0{3}^{^{\prime}^{\prime}}\mathrm{S}$ $40\textdegree 5{4}^{^{\prime}}1{8}^{^{\prime}^{\prime}}\mathrm{W}$, el. 524 m, 14–16.ii.2013, DM Takiya, JA Rafael, RR Cavichioli & APM Santos cols., Malaise trap, 5 males (MNRJ).

**Etymology.** This species is named in reference to Ubajara National Park, in the municipality with the same name, where the types were collected from.

**Remarks.** This new species appears to be a member of the *nigritta* group because of their internal pouches between abdominal segments V and VI and the presence of two long subapical spines on phallus. *Metrichia ubajara* **sp. nov.** resembles *M*. *potosina* and *M. goiana* **sp. nov.** because of the rounded and elongate inferior appendages in lateral view. However, in *M*. *ubajara* **sp. nov.** the inferior appendages lack the “tooth” mentioned for these two species. As well, *M*. *ubajara* **sp. nov.** can be recognized by the phallic apex with a broad sclerotized plate wrapping the ejaculatory duct.

### *Metrichia vulgaris* sp. nov.

urn:lsid:zoobank.org:act:BBC0387F-C111-4CA7-834A-F3711F7707F7

(Fig. 25)

**Figure 25:**
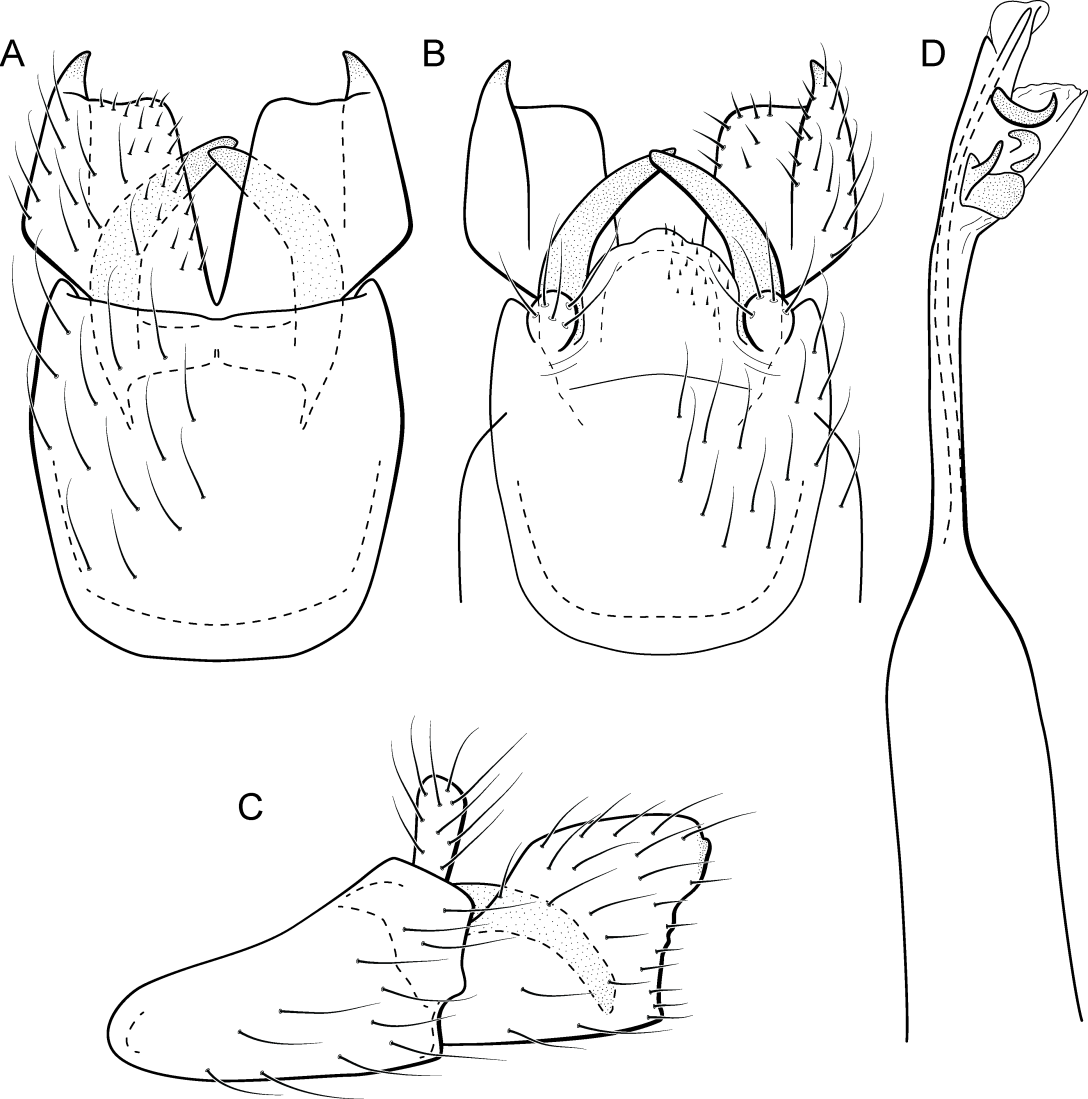
*Metrichia vulgaris* **sp. nov.**, male genitalia. (A) ventral view; (B) dorsal view; (C) lateral view; (D) phallus, dorsal view.

**Figure 26:**
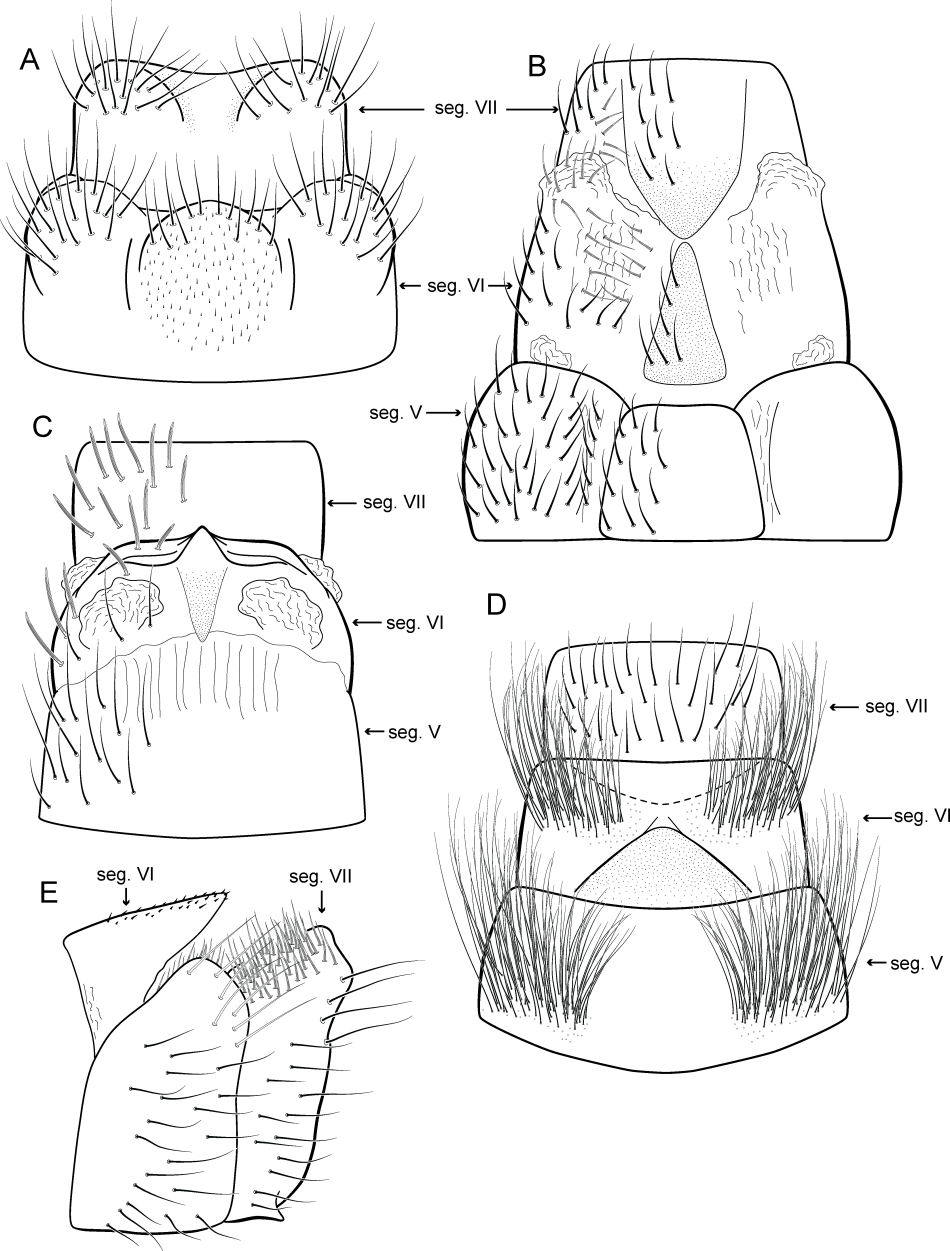
Abdominal modifications of *Metrichia* species. (A) *M. acuminata* **sp. nov.**, segments VI and VII, dorsal view; (B) *M. itabaiana* **sp. nov.**, segments V, VI, and VII, dorsal view; (C) *M. longissima* **sp. nov.**, segments V, VI, and VII, dorsal view; (D) *M. peluda* **sp. nov.**, segments V, VI, and VII, dorsal view; (E) *M. simples* **sp. nov.**, segments VI and VII, lateral view.

**Adult male.** Length 2.7–3.1 mm (*n* = 12). General color, in alcohol, brown. Head with no modifications. Ocelli 3. Antenna simple, 21-articulated. Maxillary palpus 5-articulated; labial palpus 3-articulated. Mesoscutellum with transverse suture. Metascutellum subtriangular. Anterior femur without processes. Tibial spur formula 1-3-4. Wing venation reduced in both wings. Abdominal segment VI with pair of internal pouches in posterodorsal area. Ventromesal process on segment VII present. Segment VIII shorter ventrally than dorsally. **Male genitalia**. Segment IX reduced dorsally; sternum subquadrangular (Fig. 25A); in lateral view, narrower anteriorly than posteriorly (Fig. 25C). Inferior appendage covered by long setae, apex excavated, posterodorsal margin acute and sclerotized; subtrapezoidal in ventral view (Fig. 25A); in lateral view, subretangular (Fig. 25C). Dorsal hook long and stout, almost reaching the inferior appendage apex; in lateral view, downturned (Fig. 25C). Preanal appendage elongate, rounded and bearing very long setae (Fig. 25B). Subgenital plate apparently absent. Tergum X membranous and rounded (Fig. 25B). Phallus tubular, elongate and slender, slightly constricted mesally; with two short subapical spines; apex rounded and folded; ejaculatory duct sclerotized and protruding apically (Fig. 25D).

**Holotype.** **BRAZIL: Rio de Janeiro:** Itatiaia, Rio Palmital, $22\textdegree 2{5}^{^{\prime}}3{4}^{^{\prime}^{\prime}}\mathrm{S}$ $44\textdegree 3{2}^{^{\prime}}5{2}^{^{\prime}^{\prime}}\mathrm{W}$, el. 637 m, 07.iii.2008, LL Dumas, JL Nessimian & MR de Souza cols., light trap, male (DZRJ).

**Paratypes.** Same data as holotype, 1 male (DZRJ), 1 male (MNRJ); same data, except Rio das Pedras, $22\textdegree 2{4}^{^{\prime}}3{3}^{^{\prime}^{\prime}}\mathrm{S}$ $44\textdegree 3{3}^{^{\prime}}0{8}^{^{\prime}^{\prime}}\mathrm{W}$, el. 706 m, 06.iii.2008, LL Dumas, JL Nessimian & MR de Souza cols., light trap, 4 males (MNRJ). **Ceará:** Ubajara, Parque Nacional de Ubajara, Rio Gameleira, $03\textdegree 5{0}^{^{\prime}}2{5}^{^{\prime}^{\prime}}\mathrm{S}$ $40\textdegree 5{4}^{^{\prime}}1{9}^{^{\prime}^{\prime}}\mathrm{W}$, el. 874 m, 20–22.iv.2012, F Limeira-de-Oliveira et al. cols., Malaise trap, 1 male (CZMA). **Goiás:** Alto Paraíso, Rio Bartolomeu tributary, $14\textdegree 0{7}^{^{\prime}}2{5}^{^{\prime}^{\prime}}\mathrm{S}$ $47\textdegree 3{0}^{^{\prime}}3{0}^{^{\prime}^{\prime}}\mathrm{W}$, el. 1,270 m, 22–25.iii.2013, APM Santos & DM Takiya cols., Malaise trap, 3 males (DZRJ).

**Etymology.** This new species is named in allusion to its unusually wide distribution throughout Brazil. From the Latin “vulgaris” meaning “common.”

**Remarks.** This new species belongs to the *campana* group due to their internal pouches between segments VI and VII and the two small subapical spines in phallus. *Metrichia vulgaris* **sp. nov.** shares with *M*. *campana*, *M*. *similis*, and *M*. *itabaiana* **sp. nov.** the general aspect of inferior appendages, with an excavated posterior margin. From these species, *M*. *vulgaris* **sp. nov.** can be distinguished by their inferior appendages, in lateral view, excavated but with ventral corner more rounded than acute and short and stout dorsal hook and tergum X short and rounded.

*Metrichia vulgaris* **sp. nov.** has an interesting distributional pattern, occurring in very distant localities in Southeastern, Centralwestern, and Northeastern Brazil. In addition to their geographic distance, these localities are included in very distinctive areas: encompassing three biomes (Atlantic Forest, Cerrado, and Caatinga) and four large river basins (East North Atlantic, Southeast Atlantic, São Francisco, and Araguaia-Tocantins). Barcode sequences corroborate these different populations as the same species, with K2P intraspecific divergences up to 4.8%. This is the highest intraspecific divergence found in our work, but this value is still lower than that observed among species in other caddisfly groups (Pauls et al., 2010; Zhou et al., 2011). GMYC analyses grouped sequences into two ‘species,’ but these groups were not related to their geographic distribution: one group included samples from Rio de Janeiro and Minas Gerais and the other group, samples from Rio de Janeiro, Minas Gerais, and Goiás. The broader geographical and specimen sampling of this species, which is directly associated with its higher intraspecific divergences, may explain the oversplitting by the GMYC method, as discussed by Talavera, Dincă & Vila (2013).

Although such wide distribution is not common for *Metrichia* species, other microcaddisflies can show continental distributions (e.g., *Oxyethira tica*). Because the knowledge about Neotropical microcaddisflies is very poor, this pattern may be more common than currently thought.

## Discussion

Although GMYC analysis overestimated the number of *Metrichia* species in our study (suggesting the split of *M*. *circuliforme* **sp. nov.** and *M*. *vulgaris* **sp. nov.** each into two ‘species’), COI sequences strongly corroborated species limits previously defined based on morphological features. In general, COI sequences of caddisflies show a robust ‘barcoding gap’ (Pauls et al., 2010; Zhou et al., 2011), making this molecular marker appropriate as a source of additional information to corroborate species delimitation or associations of different life stages.

Microcaddisflies are extremely diverse and poorly known, and when associated with the morphological data, the use of molecular information can result in a more robust taxonomy for this group. Although methods such ABGD and GMYC should not be used alone to determine ‘species,’ they are useful tools to identifying ‘potential species’ (Puillandre et al., 2012; Talavera, Dincă & Vila, 2013), especially in very diverse groups and/or with dubious morphology-based identification. The wide distribution of *Metrichia vulgaris* **sp. nov.** could indicate the existence of different cryptic species; however, morphology and barcode data agreed to define this group as a single species. Although GMYC overestimated the number of *Metrichia* species in our analysis, we consider this method an important tool for preliminary distinction when taxonomic information is poor.

Based on the presence of abdominal modifications, such as internal pouches, external sclerotized plates, and brushes of long setae, and features of male genitalia, six species groups have been proposed for *Metrichia* (Flint Jr, 1972; Bueno-Soria & Holzenthal, 2003). These abdominal modifications usually arise from segments V, VI, and VII, and, in general aspect, are very distinctive from each other (Fig. 26), possibly representing non-homologous structures. Nevertheless, most of the species described herein fit in these previously defined groups. Present analyses of COI sequences (Fig. 2 and Supplemental Information 4) recovered neither the monophyly of *Metrichia* nor any of the species groups tested (*aberrans*, *campana*, and *nigritta* groups). However, these relationships need to be analyzed further with better taxon and molecular marker sampling.

Herein, we have also used molecular data to associate larvae and adults of *M*. *bonita* **sp. nov.** Larvae remain unknown for most Neotropical species of Trichoptera, and they are even less known for microcaddisflies. Rearing immatures is very difficult and association based in co-occurrence with adults is not possible when several species of the same genus co-occur. In this way, DNA barcodes are a powerful tool, allowing the association and description of immature stages (Shan, Yang & Sun, 2004; Pauls et al., 2010; Ruiter, Boyle & Zhou, 2013). Barcode reference libraries for caddisflies are available for specimens from other regions (Zhou et al., 2009; Zhou et al., 2011; Ruiter, Boyle & Zhou, 2013) and represent an important source of information for taxonomic work as well as for ecological and evolutionary studies. We expect that molecular data will become increasingly common for Neotropical caddisflies as it facilitates the understanding of their diversity in this region.

## Supplemental Information

10.7717/peerj.2009/supp-1 Supplemental Information 1 Detailed list of collecting sites in Brazil where new species of *Metrichia* were found

10.7717/peerj.2009/supp-2 Supplemental Information 2 Google Earth (.kmz) file with collecting localities of new *Metrichia* species

10.7717/peerj.2009/supp-3 Supplemental Information 3 FASTA format alignment of COI sequence data of *Metrichia* and related microcaddisflies

10.7717/peerj.2009/supp-4 Supplemental Information 4 Consensus phylogram (50% majority-rule) from BI analyses of COI sequences (mean ln*L* = − 5464.29) of *Metrichia* and related microcaddisflies. Values displayed near branches are posterior probabilities

10.7717/peerj.2009/supp-5 Supplemental Information 5 Pairwise K2P divergences of COI sequences of *Metrichia* and related microcaddisflies

## Additional Information and Declarations

### Field Study Permissions

The following information was supplied relating to field study approvals (i.e., approving body and any reference numbers): Collecting permits in Brazil were issued by Instituto Chico Mendes de Conservação da Biodiversidade (ICMBio) (SISBIO 43047 and 14591).

### DNA Deposition

The following information was supplied regarding the deposition of DNA sequences: GenBank accession numbers in Table 1.

### Data Availability

The following information was supplied regarding data availability: The raw data has been supplied as a Supplemental Dataset.

### New Species Registration

The following information was supplied regarding the registration of a newly described species: The LSID for this publication is: urn:lsid:zoobank.org:pub:D8D4049E-494B-4A30-92AC-F8F42D2B54B9.
