# Supplementary material for: Integrative taxonomy of Metrichia Ross (Trichoptera: Hydroptilidae: Ochrotrichiinae) microcaddisflies from Brazil: descriptions of twenty new species
Source: PeerJ. 2016 May 5;4:e2009. doi: 10.7717/peerj.2009 (PMC4860326; doi:10.7717/peerj.2009)
Supplement: Supplemental Information 1 [file peerj-04-2009-s001.pdf]

Integrative taxonomy of *Metrichia* Ross (Trichotptera: Hydroptilidae: Ochrotrichiinae) microcaddisflies from Brazil: descriptions of twenty new species

ALLAN P M SANTOS[*]; DANIELA M TAKIYA & JORGE L NESSIMIAN

Collecting sites in Brazil where new species of *Metrichia* were found.

State	Municipality	Locality	Latitude	Longitude	Elev. (m)	River Basin	Species
Alagoas	Quebrangulo	Reserva Biológica de Pedra Talhada, Rio Caranguejo	09º15’26”S	36º25’08”W	550	Eastern North Atlantic	*Metrichia acuminata* *Metrichia talhada*
Ceará	Ubajara	Parque Nacional de Ubajara, Cachoeira do Gameleira	03º50'21"S	40º54'23"W	880	Eastern North Atlantic	*Metrichia acuminata*
Ceará	Ubajara	Parque Nacional de Ubajara, Rio das Minas	03º50’03”S	40º54’18”W	524	Eastern North Atlantic	*Metrichia acuminata* *Metrichia rafaeli* *Metrichia ubajara*
Ceará	Ubajara	Parque Nacional de Ubajara, Rio das Minas	03º49’58”S	40º53’53”W	420	Eastern North Atlantic	*Metrichia rafaeli* *Metrichia ubajara*
Ceará	Ubajara	Parque Nacional de Ubajara, Rio Gameleira	03º50’25”S	40º54’19”W	874	Eastern North Atlantic	*Metrichia vulgaris*
Goiás	Alto Paraíso de Goiás	tributary of Rio Bartolomeu	14º07’25”S	47º30’30”W	1165	Araguaia-Tocantins	*Metrichia goiana* *Metrichia itabaiana* *Metrichia vulgaris*
Mato Grosso do Sul	Bonito	Rio Formosinho	21º10’16”S	56º26’47”W	275	Paraguay	*Metrichia bonita* *Metrichia formosinha*
Minas Gerais	Catas Altas	RPPN Santuário do Caraça, Ribeirão Caraça	-	-	-	São Francisco	*Metrichia caraca*
Minas Gerais	São Roque de Minas	Parque Nacional da Serra da Canastra, Fazenda Velha, Córrego dos Pombos	20º14’57”S	46º38’05”W	997	São Francisco	*Metrichia caraca*
Minas Gerais	Jaboticatubas	Parque Nacional da Serra do Cipó, Cachoeira da Farofa	19º22’47”S	43º34’36”W	811	São Francisco	*Metrichia farofa*

Minas Gerais	Jaboticatubas	Parque Nacional da Serra do Cipó, Ribeirão Mascates	19º24’02”S	43º34’35”W	820	São Francisco	*Metrichia farofa*
Paraná	Céu Azul	Parque Nacional do Iguaçu, Rio Azul	25º09’21”S	53º47’44”W	510	Paraná	*Metrichia azul*<br>*Metrichia forceps*<br>*Metrichia simples*
Rio de Janeiro	Angra dos Reis	Rio Bracuí	23º00’23”S	44º29’15”W	75	Southeast Atlantic	*Metrichia bracui*
Rio de Janeiro	Itatiaia	Parque Nacional do Itatiaia, Córrego do Maromba	22º25’32”S	44º37’03”W	1250	Southeast Atlantic	*Metrichia bracui*
Rio de Janeiro	Itatiaia	Rio das Pedras, Cachoeira de Deus	22º25'00"S	44º32'50"W	689	Southeast Atlantic	*Metrichia circuliforme*
Rio de Janeiro	Itatiaia	Rio das Pedras	22º24’33”S	44º33’08”W	706	Southeast Atlantic	*Metrichia circuliforme*<br>*Metrichia curta*<br>*Metrichia vulgaris*
Rio de Janeiro	Itatiaia	Parque Nacional do Itatiaia, Córrego Simon	22º26’16”S	44º36’20”W	1033	Southeast Atlantic	*Metrichia circuliforme*
Rio de Janeiro	Itatiaia	Rio Palmital	22º25’34”S	44º32’52”W	637	Southeast Atlantic	*Metrichia longissima*<br>*Metrichia peluda*<br>*Metrichia vulgaris*
Rio de Janeiro	Teresópolis	Parque Nacional da Serra dos Órgãos, Rio Paquequer	22º27’25”S	42º59’52”W	1100	Southeast Atlantic	*Metrichia longissima*<br>*Metrichia tere*
Rio de Janeiro	Itatiaia	1st order tributary of Rio Palmital	22º25’40”S	44º32’46”W	584	Southeast Atlantic	*Metrichia peluda*
Sergipe	Areia Branca	Parque Nacional da Serra de Itabaiana, Rio dos Negros	10º44’51”S	37º20’24”W	208	Eastern Atlantic	*Metrichia itabaiana*
Sergipe	Areia Branca	Parque Nacional da Serra de Itabaiana, Riacho Água Fria	10º45’17”S	37º20’32”W	196	Eastern Atlantic	*Metrichia itabaiana*
