# Supplementary material for: Integrative taxonomy of Metrichia Ross (Trichoptera: Hydroptilidae: Ochrotrichiinae) microcaddisflies from Brazil: descriptions of twenty new species
Source: PeerJ. 2016 May 5;4:e2009. doi: 10.7717/peerj.2009 (PMC4860326; doi:10.7717/peerj.2009)
Supplement: Supplemental Information 4 [file peerj-04-2009-s004.pdf]

Integrative taxonomy of *Metrichia* Ross (Trichotptera: Hydroptilidae: Ochrotrichiinae) microcaddisflies from Brazil: descriptions of twenty new species

ALLAN P M SANTOS*; DANIELA M TAKIYA & JORGE L NESSIMIAN

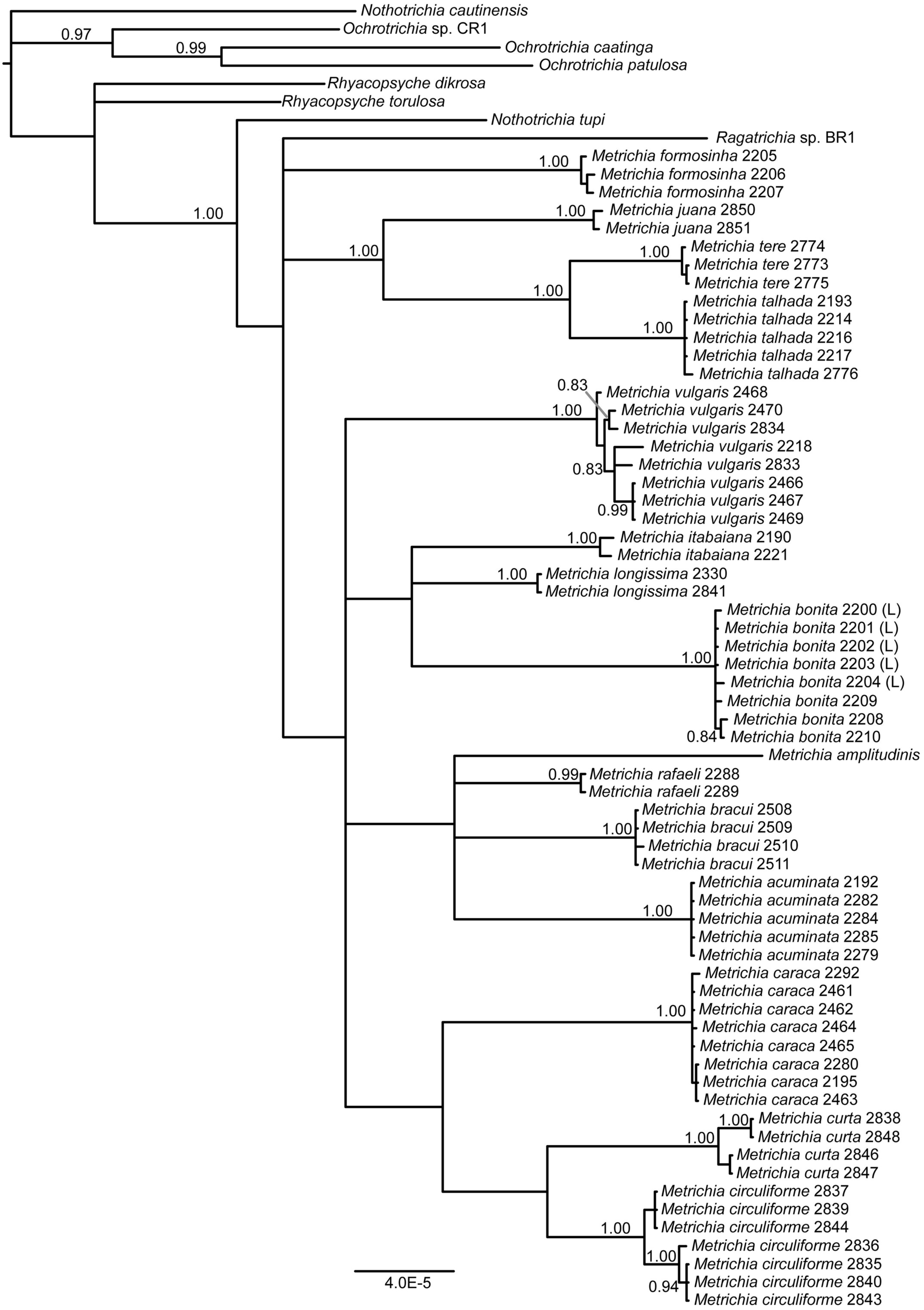

Consensus phylogram (50% majority-rule) from BI analyses of COI sequences (lnL = -5464.29). Values displayed near branches are posterior probability.
